# Goodness of Fit in the Marginal Modeling of Round-Trip Times for Networked Robot Sensor Transmissions

**DOI:** 10.3390/s25175413

**Published:** 2025-09-02

**Authors:** Juan-Antonio Fernández-Madrigal, Vicente Arévalo-Espejo, Ana Cruz-Martín, Cipriano Galindo-Andrades, Adrián Bañuls-Arias, Juan-Manuel Gandarias-Palacios

**Affiliations:** Systems Engineering and Automation Department, Institute for the Research in Mechatronic Engineering and Cyberphysical Systems, University of Málaga, 29004 Málaga, Spain; jafernandez@uma.es (J.-A.F.-M.); varevalo@uma.es (V.A.-E.); cga@uma.es (C.G.-A.); adrianb_arias@uma.es (A.B.-A.); jmgandarias@uma.es (J.-M.G.-P.)

**Keywords:** goodness of fit, hypothesis test, round-trip times modeling, networked robots

## Abstract

When complex computations cannot be performed on board a mobile robot, sensory data must be transmitted to a remote station to be processed, and the resulting actions must be sent back to the robot to execute, forming a repeating cycle. This involves stochastic round-trip times in the case of non-deterministic network communications and/or non-hard real-time software. Since robots need to react within strict time constraints, modeling these round-trip times becomes essential for many tasks. Modern approaches for modeling sequences of data are mostly based on time-series forecasting techniques, which impose a computational cost that may be prohibitive for real-time operation, do not consider all the delay sources existing in the sw/hw system, or do not work fully online, i.e., within the time of the current round-trip. Marginal probabilistic models, on the other hand, often have a lower cost, since they discard temporal dependencies between successive measurements of round-trip times, a suitable approximation when regime changes are properly handled given the typically stationary nature of these round-trip times. In this paper we focus on the hypothesis tests needed for marginal modeling of the round-trip times in remotely operated robotic systems with the presence of abrupt changes in regimes. We analyze in depth three common models, namely *Log-logistic*, *Log-normal*, and *Exponential*, and propose some modifications of parameter estimators for them and new thresholds for well-known goodness-of-fit tests, which are aimed at the particularities of our setting. We then evaluate our proposal on a dataset gathered from a variety of networked robot scenarios, both real and simulated; through >2100 h of high-performance computer processing, we assess the statistical robustness and practical suitability of these methods for these kinds of robotic applications.

## 1. Introduction

The correctness of computing systems that are distributed over a network depends directly on dealing with time, in particular on their capabilities for both synchronizing clocks that tick with different frequencies and offsets [1] and dealing with the delays involved in their exchange of information [2]. Regarding the latter, there are a number of methods that model statistically those delays, but many of them impose requirements that do not fit well the problem of sensory transmissions in networked robots [3]. These particular scenarios demand online processing of the measured times, as opposed to offline approaches; they must account not only for network latency but also for delays introduced by software; they are required to provide rich information about the behaviour of RTTs, and they usually have no mathematical model available for the dynamics of the system (plant) to be controlled.

In that kind of setting, one or more robots need to transmit data (the bulk comprising sensory data) to remote computers that process them and make decisions to be executed on board. This forms a control loop [4] where each iteration takes a stochastic time, referred here to as *round-trip time* (RTT) and may affect the stability and quality of the robot’s performance.

Notice that in other communities, there are diverse definitions of RTT. In our context that time comprises both hardware (network + sensors + computers) and software (drivers + operating systems + applications) delays, all of these components provide some part of the total cycle time, and, in most cases, none of them satisfy hard real-time requirements [5]. Nevertheless, even a non-deterministic, probabilistic modeling of these round-trip times is important for the correct operation of the robot, e.g., for assessing the probability that the next round-trip time lies within some interval.

In this paper we deal with marginal modeling of these round-trip times aimed at providing a reasonable computational cost for fully or at least batched online processing, which provides more information about the RTT sequence than just the expected next measurement, and a simplified representation of the gathered RTTs as close as possible to the actual data.

Marginal modeling, by definition, is carried out based on particular probability distributions. Diverse distributions have been proposed for network transmissions: The *Exponential* model, which is a general and common representation of independent inter-arrival times, is useful for fast communications, for instance, among networked sensors [6] and also as a limiting distribution when the load in the network increases, since it is closely related to Poisson processes [7]. The *Log-normal* distribution is used for modeling the traffic in wired networks (LAN) [8], which provides more flexibility than the exponential through an additional parameter, and other distributions as well, such as *Weibull* (which generalizes the *Exponential*), *Erlang* (a product of *Exponentials*), and *Gamma* (a further generalization of the *Exponential*) [9]. Remarkable flexibility in a marginal modeling context has been shown by the *Log-logistic* model [10], mainly due to its heavy tail and variety of shapes.

In addition, a number of general change detection methods that can be applied to the detection of abrupt regime changes in the RTT sequence exist in the literature [11,12,13]. However, many of these (i) require access to future data windows, leading to additional delays in detection—; (ii) do not offer rigorous statistical guarantees; or (iii) do not assume a particular parametric distribution of the data that carries out enough information about the RTTs and converge to the correct model with short samples.

On the other hand, performing a goodness-of-fit hypothesis test is a rigorous approach [14,15] that, through the rejection or not of the hypothesis that the current distribution explains the last round-trip times, serves to detect changes in regimes, and it works without knowing any further data and provides more accurate modeling due to the assumption of a particular, well-defined probability distribution. Elsewhere we have applied it to the case of the *Log-logistic* distribution [10], but without a thorough analysis of its statistical performance.

Here we study in depth the use of goodness-of-fit hypothesis tests for the main marginal probability distributions used in similar problems. Note that the networked robot context has certain particularities that define special requirements not always found in other studies:

- To have a location parameter in all these distributions.
- To use suitable ranges of all their parameters.
- To consider possible numerical errors in the computations.
- To employ parameter estimation procedures adapted to all of that.
- To have a rigorous assessment of the performance of the goodness-of-fit tests, in particular their significance and power, under those conditions.

This lead to some modifications in the classical tests in order to preserve their significance and power as much as possible. Once assessed, these tests can be applied to the detection of changes in regimes using the following null hypothesis:

**$H_{0}$:**  *The current sample of the sequence of round-trip times has been generated by a given form of (current distribution model).*

In the case the hypothesis is rejected, a change of regime can be assumed. The sample can grow as long as new round-trip times that do not reject the hypothesis are added, possibly re-estimating the parameters of the distribution along the way.

For this work we have used an extensive dataset of round-trip times collected in diverse networked robot scenarios [16]. It has been served both for defining the suitable parameter ranges for the distributions and for assessing the performance of both the parameter estimation and goodness-of-fit procedures. Our results confirm that particular adjustments on existing methods are needed for this kind of systems; we also report comparisons between the studied distributions.

In summary, the main contributions of this paper are as follows:

- New modified Maximum Likelihood Estimators for the three probability distributions that are common in marginal modeling of network communications, which are aimed at coping with the particular issues found in the context of networked robots’ round-trip times modeling.
- A statistical evaluation of the effects of the discretization of time performed by computers in the estimation of parameters of those continuous distributions.
- Novel Monte Carlo estimates of the thresholds corresponding to a significance level of 0.05 for the well-known Cramér-von Mises goodness-of-fit test in the case where the parameters of those distributions are estimated from the sample using our modified MLEs.
- Smooth polynomial interpolations of the obtained thresholds that enable compact and relatively fast calculations with any sample size.
- A statistical assessment of the significance and power of the GoF tests with the provided thresholds.
- Experimental evaluation of the use of our tests for modeling both real and synthetic sequences of RTTs in networked robot applications, including their computational cost, their online modeling capacities, and their ability to detect abrupt changes in regimes.

The rest of the document is structured as follows: In Section 2 we summarize the main trends dealing with stochastic RTTs in the literature; in Section 3 we provide the formal definitions of the three analyzed models; in Section 4 we describe the procedures to estimate their parameters from round-trip samples, and in Section 5 we study the hypothesis tests devised for them. In Section 6 we evaluate the performance of our procedures on the dataset. Finally, we include the main conclusions and future work.

## 2. Related Work

In the following section we classify the main RTT characterization methods that can be found in the literature, though the specific goals can differ from one work to another depending on the context.

Communication networks are the main community dealing with round-trip times, which are a central metric in performance evaluations and usually comprise the network components only. Their specific definition and therefore the techniques used to model them mostly depend on the layer of the communication stack where they are measured—e.g., at the low levels of the ISO stack, which is close to the network hardware, RTTs can be modeled and predicted with the methodologies listed in [17].

Statistics offers a set of simple and powerful techniques to model and predict round-trip times. For example, there is a well-known statistical expression that estimates RTT occurring in the TCP protocol, namely the Exponentially Weighted Moving Average (EWMA) [18]:

(1) $${\hat{x}}_{t}=\left(1-\alpha \right)*{\hat{x}}_{t-1}+\left(\alpha \right)*x_{t},$$

where $x_{t}$ is the current measured RTT and ${\hat{x}}_{t}$ the estimated one, computed over time. The parameter α is usually assigned a value of one-eighth, according to RFC 6298 [19], which leads to certain loss of adaptability. In general, the EWMA is a simple methodology and can be run fully online, but since it acts as a smoothing filter, it does not provide fast detection of abrupt changes in the RTT values nor a complete model from which other deductions can be drawn (e.g., what is the probability of the next RTT lying in a given interval).

With the goal of analyzing the end-to-end latency in different geographical areas of the world and its implications on networking services, ref. [20] uses linear regression models—seeking the RMSE in the regression lines—based on RTT data collected worldwide through a span of several years. These are implemented in ML libraries written in the most common programming languages. However, their approach is completely offline and does not consider bursts or regime changes in the transmission; furthermore, their definition of round-trip time does not include the access from the user computer to the network.

Another statistical approach is the one where [21] applies to industrial environments, with the aim of minimizing the impact of undesired delays in the performance of the control loops of a plant. The authors of [21] use an ARIMA-based solution to model and then predict (point prediction) the network delays; this prediction is then combined with the use of specific algorithms that help to manage the effects of those delays in the loops. ARIMA prediction of network traffic is still a matter of research, particularly if hybridized with neural networks [22,23]; however, this is an offline approach that requires a large amount of training time and data.

The work in [24] focuses on the congestion control at the TCP level. The authors have designed a CUSUM Kalman Filter, an adaptive filter that detects abrupt changes in RTT. This filter needs a basic model of the network nodes, whereas our approach does not require any previous model. Again, recent research also tends to hybridize CUSUM with neural networks, though it is mainly applied to the detection of abnormal traffic conditions in communication networks [25]. A different congestion control strategy for mobile networks is the one in [26], which uses a two-stage Kalman filter plus an adaptive clipping phase but also depends on the availability of a network model.

Closely related to Kalman Filters, Hidden Markov Models (HMMs) [27] are another option for RTT estimation. They are able to grasp the time dependencies in a sequence of time measurements and abrupt changes in regimes, but these benefits come at the expense of a significant computing cost for training the model. In particular, ref. [28] uses a Hierarchical Dirichlet Process HMM (HDP-HMM) to predict RTTs in the long term with a precise identification of changes and bursts in the signal. The model has a computation time of 5 s; thus it has to be recalculated at regular intervals; i.e., it works in batches of RTTs. In a robotics application like ours, the robot would work in an open loop from the previous model’s update for at least those 5 s, which can lead to a dangerous situation if in the meantime there is a downgrading or regime change in the transmissions. Other recent works on the use of HMMs in communication networks exist, but they usually deal with detecting abnormal conditions rather than modeling/forecasting transmission times [29].

Recently there is an important trend to use machine learning techniques that, in general, provide fewer guarantees but better adjustment to complex situations for modeling round-trip times. In [30] the RTT is estimated using Regularization Extreme Machine Learning (RELM), which combines the training speed of ELM networks without overfitting issues thanks to the regularization part of machine learning. It obtains accurate RTT estimations with good time performance in the validation and testing phases, but there are no results of a real implementation that deals with bursts or regime changes. There are also deep learning approaches for RTT estimation. In this area, the authors of [31] propose a recurrent neural network (RNN) with a minimal gated unit (MGU) structure in order to predict RTTs; this solution obtains very good results when compared with some other RTT modeling techniques; however, it does not present information about the power and time resources needed neither for the initial offline RNN training phase nor for the performance of RTT prediction on the fly. The work in [32] presents a DL classic strategy with a LSTM-based RNN architecture that passively predicts RTT in an intermediate node (therefore not in the transmission endpoints, as seen in our approach) that can be trained on an experimental testbed and then delivered without accuracy loss to a real environment; i.e., it shows good transfer learning capabilities. However, again there is no information about the costs of the training phase, and there are no real tests with abrupt changes in the transmission time. Also working on the modeling and prediction of RTT at the TCP level, ref. [33] reports a Transformer-based solution in a preliminary stage but with good results. As in previous cases, that demands a previous training phase of which there is no information about its time and power costs, and its performance in a real setting with bursts and changes in regimes is unknown too. Notice that these approaches mostly provide a prediction of the next round-trip time only.

Most of the mentioned methods address the dependencies existing between successive round-trip times and try to model these dependencies as accurately as possible, usually without achieving complete online performance in a real system and in many cases without explicitly handling abrupt changes in regimes in the signal. A different approach is to drop those dependencies and deal with the data as though they were *iid*, i.e., samples drawn from some marginal probability distribution. As long as abrupt changes in regimes in the sequence of round-trip times are detected, and different models are fitted to each regime, this approximation may provide good results with less computational costs than other methods, and since they are based in particular probability distributions, they also serve to not only draw different conclusions from the data but also to point value forecasting; they can also be adapted to online estimation quite easily. The main reason for their good behavior is that the round-trip times in real systems have little trends, as they are composed in most cases of segments of different characteristics that are stationary.

## 3. Model Definitions

In this section we define the three probability distributions used to model round-trip times marginally in this work: the *Exponential*, *Log-normal*, and *Log-logistic* distributions. For each of them, we summarize their cumulative and density functions (with a location parameter), along with the expressions of their statistical moments, which are later used in parameter estimation and goodness-of-fit testing.

### 3.1. Exponential Distribution

The (shifted) *Exponential* distribution is defined by the following distribution and density distribution functions, respectively:

(2) $$\begin{array}{cc} F(x;\alpha ,\beta ) & =1-e^{-(x-\alpha )/\beta },\\ f(x;\alpha ,\beta ) & =\frac{1}{\beta }e^{-(x-\alpha )/\beta }, \end{array}$$

where for x≥α (otherwise the functions are 0), α>0 is the location parameter (we lower the bound to 0 to avoid null transmission times) and β>0 is its shape. Its expectation is α+β, and its variance is $\beta^{2}$, with both moments always being well defined.

### 3.2. Log-Normal Distribution

The *Log-normal* distribution describes a random variable whose logarithm follows a *normal* distribution; when generalized to include a location γ, it is defined as

(3) $$\begin{array}{cc} F(x;\gamma ,\mu ,\sigma ) & =\Phi \left(y\right),\;y=\frac{ln(x-\gamma )-\mu }{\sigma },\;\Phi \left(y\right)=\frac{1}{2\pi }\int_{-\infty }^{y}e^{-t^{2}/2}dt,\\ f(x;\gamma ,\mu ,\sigma ) & =\frac{1}{\left(x-\gamma \right)\sigma \sqrt{2\pi }}e^{-\frac{y^{2}}{2}}, \end{array}$$

where for x>γ, Φ is the function of a standard normal distribution (i.e., with an expectation of 0 and a variance of 1), γ≥0 is the location (we allow an equality because this distribution cannot generate values exactly at its location), μ is the expectation, and σ>0 is the standard deviation of the logarithm of the values. The expectation of the *Log-normal* distribution is $\gamma +exp(\mu +\sigma^{2}/2)$, and its variance is $exp\left(2\mu +\sigma^{2}\right)\cdot \left(exp\left(\sigma^{2}\right)-1\right)$, with both always well defined. The third moment (skewness) is also needed for parameter estimation, as we discuss later on in Section 4.2; it is formally defined as $exp\left(3\mu +3\sigma^{2}/2\right)\cdot \left(exp\left(\sigma^{2}\right)-1\right)\cdot \left(exp\left(\sigma^{2}\right)+2\right)$.

### 3.3. Log-Logistic Distribution

The *Log-logistic* distribution corresponds to a random variable whose logarithm has a *Logistic* distribution; it is defined, including location *a*, as follows:

(4) $$\begin{array}{cc} F(x;a,b,c) & =\frac{1}{1+y},\;y={\left(\frac{x-a}{b}\right)}^{-1/c},\\ f(x;a,b,c) & =\frac{y}{c\left(x-a\right){\left(1+y\right)}^{2}}, \end{array}$$

where a≥0 is the location, b>0 is the scale, and c>0 is the shape. In this case, the first moments do not always exist; for the expectation to be defined, c<1 must be satisfied, while for the variance, the condition is c<1/2. Since having well-defined moments is useful when working with marginal modeling (e.g., for prediction) and for c<0.05 the shape becomes too close to a *normal* distribution to justify the use of a *Log-logistic* distribution (and easily leads to overflows in the calculations), we constrain c∈[0.05,0.5).

Within that range, the expectation is a+bρ/sin(ρ) and the variance is $b^{2}\left(2\rho /sin\left(2\rho \right)-{\left(\rho /sin\left(\rho \right)\right)}^{2}\right)$, where ρ=πc. These formulae can also be rewritten using the gamma function, where $\Gamma \left(z\right)=\int_{0}^{\infty }t^{z-1}e^{-t}dt$, and its reflection formula, where Γ(1−x)Γ(x)=π/sin(πx), and its fundamental property Γ(1+x)=xΓ(x) lead to ρ/sin(ρ)=β(1+c,1−c), with β(m,n)=Γ(m)Γ(n)/Γ(m+n) being the *beta* function; therefore, for instance, the expectation of the *Log-logistic* distribution can also be written as a+b·β(1+c,1−c), which can be convenient because there exist libraries that provide better numerical behavior for this form (although with slower computations).

## 4. Parameter Estimation

In a real setting, where we are measuring round-trip times and trying to model them with some of the distributions defined in Section 3, we need procedures to estimate the parameters of those distributions from the sample. Since we are considering all round-trip times in the sample as *iid* in this marginal approach, we are able to use Maximum Likelihood Estimators (MLEs), in particular their *log-likelihood* form, because our models have exponential terms. This kind of estimator has good properties under mild conditions, such as minimum variance and asymptotic efficiency [34], and with minor modifications, they can be unbiased.

There are two particular issues in the context of networked robots, regarding computation on the measured RTTs, that deserve some comment:

- Round-trip times are measured as discrete values, for instance, in milliseconds or in nanoseconds in the case of higher-resolution systems. The estimation procedures may be affected by this since the distributions we use are continuous, but using discrete ones would increase the computational cost considerably; thus we proceed with the continuous models. In Section 6.2 we include a more in-depth analysis of this issue.
- In some scenarios, the location of the distribution may be large; if the variance is small, accuracy problems may arise when using a floating-point representation due to the amount of digits needed both in the integral and in the fractional part of the numbers, and round-off errors may accumulate. We carried out the same experiments described further on with a previous shifting of all the RTTs of the samples towards 0.1 to address this issue (the original sample location can be easily recovered later just by performing the opposite shift on the location estimate). However we obtained much better results by leaving the sample unshifted, probably because measuring RTTs in milliseconds (integer parts are not too long) does not force the floating-point representation near its limits; Additionally, keeping this representation instead of a fixed point allows us to be as little invasive as possible in existing robotic software, leverages its wide hardware support in general-purpose computers, and provides greater flexibility to adapt to the diverse number scales found in our dataset.

In the following section, we describe the parameter estimation procedures for each of the three probability distributions considered in this work. We propose that some variations are needed to cope with the particularities of our networked robot context; thus the properties of the goodness-of-fit tests that are based on these estimators have varied as well; new thresholds for their correct work are deduced in Section 5.

### 4.1. Exponential Distribution

We begin by deriving the MLE for the *Exponential* distribution. If the round-trip-time sample *X* consists of *n* measurements $\left\{x_{i}\right\}$, it is easy to deduce the theoretical MLE for the parameters of this distribution:

(5) $$\begin{array}{cc} L(X;\alpha ,\beta ) & =\prod_{i=1}^{n}\frac{1}{\beta }e^{-\frac{xi-\alpha }{\beta }}=\frac{1}{\beta^{n}}e^{-\frac{\sum_{i=1}^{n}\left(x_{i}-\alpha \right)}{\beta }},\\ LL(X;\alpha ,\beta ) & =ln\left(L\right)=-nln\beta -\frac{\sum_{i=1}^{n}\left(x_{i}-\alpha \right)}{\beta },\\ \frac{\partial (LL)}{\partial \alpha } & =\frac{n}{\beta }>0,\\ \frac{\partial (LL)}{\partial \beta } & =\frac{1}{\beta^{2}}\left(\sum_{i=1}^{n}\left(x_{i}-\alpha \right)-n\beta \right),\\ \frac{\partial (LL)}{\partial \beta } & =0\Rightarrow \begin{array}{c} \hat{\beta }=\frac{1}{n}\sum_{i=1}^{n}\left(x_{i}-\alpha \right) \end{array}, \end{array}$$

where we assume that α≥0 and β>0. As seen in the equations, the derivative for α cannot be zeroed; we must maximize instead the log-likelihood LL (with respect to α); since the derivative is always positive, LL is monotonically increasing with α; therefore, in order to maximize LL, we just minimize the term $\sum_{i=1}^{n}\left(x_{i}-\alpha \right)$, and since $x_{i}\geq \alpha$ for all $x_{i}$, this can be achieved with $\begin{array}{c} \hat{\alpha }=min\left(x_{i}\right) \end{array}$. Now, this estimate $\hat{\alpha }$ is the only available value for α; thus by applying the plug-in principle, we use it in the calculation of $\hat{\beta }$.

Unfortunately, there is a non-zero probability that the minimum of any finite sample drawn from the distribution is *not equal* to the true α (particularly considering the round-trip time discretization performed by the computer); therefore both $\hat{\alpha }$ and $\hat{\beta }$ are biased. In [35] the following unbiased estimates are provided:

(6) $$\begin{array}{cc} \hat{\beta } & =\frac{n}{n-1}\left(\frac{1}{n}\sum_{i=1}^{n}x_{i}-min\left(X\right)\right),\\ \hat{\alpha } & =min\left(X\right)-\frac{\hat{\beta }}{n}. \end{array}$$

However, in our case, these estimates present a drawback as well: when the average of the sample is equal to or greater than n·min(X), the unbiased estimate $\hat{\alpha }$ of Equation (6) may be zero or negative—which would invalidate the model in our setting. This can occur when there are large values in the sample, such as those far to the right of the distribution support, distorting the mean up to that point.

The variation we propose to the method in Equation (6) is to employ Equation (6) and then check whether $\hat{\alpha }\leq 0$; if that occurs, we fall back to the biased estimate of Equation (5).

### 4.2. Log-Normal Distribution

The second distribution to provide estimators for the model is the *Log-normal* distribution. Estimating its parameters boils down to estimating its location (γ), since after that we can transform the sample $X=\{x_{i}\}$ into another one $Y=\{ln\left(x_{i}-\gamma \right)\}$ that comes from N(x;μ,σ) and then calculate the MLE estimates for that *normal* distribution through the well-known average and standard deviation of the sample, where

(7) $$\begin{array}{cc} \hat{\mu } & =\frac{1}{n}\sum_{i=1}^{n}y_{i},\\ \hat{\sigma } & =\sqrt{\frac{1}{n-1}\sum_{i=1}^{n}{\left(y_{i}-\hat{\mu }\right)}^{2}}. \end{array}$$

Despite its apparent simplicity, the problem of estimating the location of a *Log-normal* distribution is a hard one—even harder than for the *Log-logistic* distribution. Over the years, several proposals for that have been reported [36,37,38,39,40], all with their pros and cons. We selected the first Cohen’s modified method [37] because of its extended use and reasonable computational cost.

The main problem of Cohen’s method is that in the scenarios of our dataset, it has shown a high probability of failing to provide estimates. To cope with that, we propose an extension to complement it with a method of moments in those cases, although that somewhat reduces the quality of the obtained estimates and affects the power of the goodness-of-fit test, as we also show later on in Section 6.1. Other methods, like [39], always provide solutions, but they are computationally prohibitive in our context.

The first modified Cohen’s method consists of finding the zero-crossings of the function $\Theta_{1}\left(\gamma \right)$ defined below (we call it the *Cohen function* from now on) and selecting the estimate $\hat{\gamma }$ from them:

(8) $$\begin{array}{cc} \Theta_{1}\left(\gamma \right) & =ln\left(x_{r}-\gamma \right)-v-k_{r}\sqrt{w-v^{2}},\\ v & =\frac{1}{n}\sum_{i=1}^{n}ln\left(x_{i}-\gamma \right),\\ w & =\frac{1}{n}\sum_{i=1}^{n}ln^{2}\left(x_{i}-\gamma \right),\\ x_{r} & =r\text{-}th\;\mathrm{value}\;\mathrm{in}\;\mathrm{the}\;\mathrm{ordered}\;\mathrm{sample},\\ k_{r} & =\Phi^{-1}\left(\frac{r}{n+1}\right). \end{array}$$

This method finds a *local* maximum of the likelihood (a global maximum exists at $\hat{\gamma }=min\left(X\right)$, $\hat{\mu }=-\infty$, ${\hat{\sigma }}^{2}=\infty$, which is useless). We instantiate the method for r=1; i.e., we use the first-order statistic (the minimum) of the sample.

In our case this MLE is constrained to $\hat{\gamma }\in \left(0,min\left(X\right)\right)$. If several zero-crossings are found, Cohen recommends taking the one that makes $\hat{\mu }$ closest to the average of *Y*, but, according to [37], our choice of r=1 and the fact that most real scenarios of round-trip time measurements that we explored admit models that are quite skewed lead to a very low probability of finding more than one optimum in this process.

**Figure 1:**
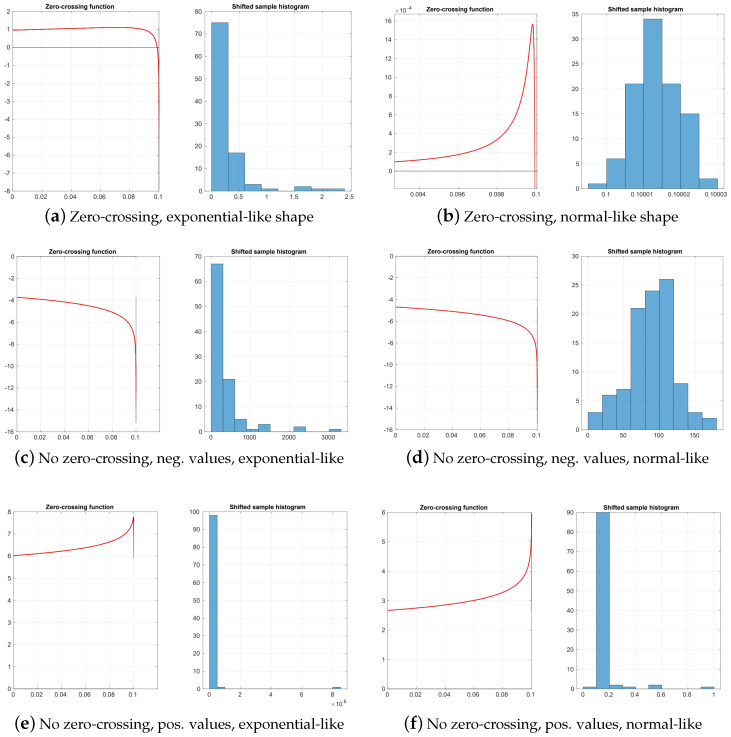
Classes of failures of the zero-crossing function found in our experiments for *Log-normal* distribution estimation, depending on their shape (more *Gaussian* or more *Exponential*). In these examples the samples were shifted first to the left to start at 0.1 in order to provide easier comparisons. The red curves are the Cohen function, the vertical black lines indicate the minima of the samples and the horizontal black lines the zero value.

In our experiments, however, we found many cases where the function $\Theta_{1}$ does not cross the abscissa, and therefore the estimation fails (examples of their typologies are shown in Figure 1). In these no-crossing cases, the value of the function at the bounds of the search region has the same sign—this suggests that a bisection algorithm [41] is the method of choice to find the zero-crossing, if there is any, since that must happen when both signs differ due to Bolzano’s theorem [42].

When the Cohen function fails, we resort to a method of moments, as explained when introducing this extension. First, we calculate the first three moments of the sample (unbiased), where

(9) $$\begin{array}{cc} \mu_{X} & =\sum_{i=1}^{n}x_{i},\\ \sigma_{X}^{2} & =\frac{1}{n-1}\sum_{i=1}^{n}{\left(x_{i}-\mu_{X}\right)}^{2},\\ s_{X} & =\frac{n}{\left(n-1)(n-2\right)}\sum_{i=1}^{n}{\left(x_{i}-\mu_{X}\right)}^{3}. \end{array}$$

Then, we minimize the 2-norm of the three-parameter vector function that represents the difference between the sample moments and the theoretical moments, where

(10) $$\begin{array}{cc} f_{1}\left(\gamma ,\mu ,\sigma \right) & =\gamma +e^{\mu +\frac{\sigma^{2}}{2}}-\mu_{X},\\ f_{2}\left(\gamma ,\mu ,\sigma \right) & =e^{2\mu +\sigma^{2}}\cdot \left(e^{\sigma^{2}}-1\right)-\sigma_{X}^{2},\\ f_{3}\left(\gamma ,\mu ,\sigma \right) & =e^{3\mu +\frac{3\sigma^{2}}{2}}\cdot \left(e^{\sigma^{2}}-1\right)\cdot \left(e^{\sigma^{2}}+2\right)-s_{X}. \end{array}$$

This is conducted through the *trust-region reflective* method for non-linear optimizations [43], with initial guesses of $\gamma_{0}=min\left(X\right)-10^{-9}$, $\mu_{0}=avg\left(ln\left(X-\gamma_{0}\right)\right)$, and $\sigma_{0}=std\left(ln\left(X-\gamma_{0}\right)\right)$. The bounds for the search are $\gamma \in [10^{-9},min\left(X\right)-10^{-9}]$, μ∈(−∞,+∞), and σ∈[ϵ,+∞), with ϵ being the minimum resolution of the floating-point computations.

This optimization may fail as well (no minimum found), but the probability of that is very low. When an estimate $\left(\hat{\gamma },\hat{\mu },\hat{\sigma }\right)$ is found, we keep only the location, $\hat{\gamma }$, and deduce the other estimates through the transformation to the *normal* distribution, since we observed better models by following that procedure.

The overall method for estimating the location of the *Log-normal* distribution is shown in pseudocode in Algorithm 1.
**Algorithm 1** Estimation of γ for the *Log-normal* distribution  **function** EstimateGamma($X=\langle x_{1},x_{2},\ldots ,x_{n}\rangle$)      S←sort(X)      r←1      n←|S|      $k_{r}\leftarrow \Phi^{-1}\left(r/\left(n+1\right)\right)$      $tol\leftarrow 10^{-9}$▹ tolerance on the RTT measurements      **if** $sign\left(\Theta_{1}\left(tol\right)\right)=sign(\Theta_{1}\left(s_{1}-tol\right)$ **then**          $\hat{\gamma }\leftarrow$MethodOfMoments(S,tol)▹ fall back to the method of moments      **else**          $\hat{\gamma }\leftarrow BisectionMethod\left(\Theta_{1},tol,s_{1}-tol\right)$▹ bisection on Cohen’s MMLE-I      **end if**  **end function**

### 4.3. Log-Logistic Distribution

Finally, we address the *Log-logistic* distribution. Its three parameters are estimated via the MLE method proposed in [44]. This involves the minimization of the 2-norm of a three-dimensional vector function with one equation per parameter, which must be performed numerically. Initial guesses for *a* (location) and *b* (scale) can be directly provided: the former should be close to the minimum of the sample, while the latter should be close to the median of the distribution once the location is taken into account, since med(X)=b when a=0; however, guessing an initial value for *c* (shape) is more challenging and requires an additional minimization procedure, which we introduce in the following section as a variation in this MLE.

The complete estimation consists of sequentially solving two non-linear, bounded optimization problems:

1. First, we compute an initial guess of *c* through the method of moments based on the fact that E[Log-logistic]=a+b·β(1+c,1−c), as explained in Section 3. We use numerical minimization with a value of *a*, which was calculated as in the *Exponential* distribution (other values slightly below the minimum provide similar results); the value for *b* is based on the median, where $\bar{b}=max\left(median\left(X-\bar{a}\right),10^{-6}\right)$, and we start at an initial c=0.05. The uni-dimensional function to minimize here is $h\left(c\right)={\left(\bar{a}+\bar{b}\cdot \beta \left(1+c,1-c\right)-avg\left(X\right)\right)}^{2}$, within the bounds c∈[0.05,0.5), which may have the minimum at one of the extremes or in the middle, which is at a zero-crossing.
2. Second, using the tuple $\left(\bar{a},\bar{b},\bar{c}\right)$ as an initial guess for the second stage, we refine the estimate of the three parameters by minimizing the 2-norm of the vector function formed by the three partial derivatives of the log-likelihood distribution (Equation (11)). The lower bounds are (ϵ,ϵ,0.05), and the upper bounds are $\left(min\left(X\right)-10^{-9},\infty ,0.5\right)$.

(11) $$\begin{array}{cc} \frac{\partial LL(a,b,c)}{\partial a}= & \sum_{i=1}^{n}\frac{\left(1+\frac{1}{c}\right)-\frac{2b^{1/c}}{c\left({\left(x_{i}-a\right)}^{1/c}+b^{1/c}\right)}}{x_{i}-a},\\ \frac{\partial LL(a,b,c)}{\partial b}= & \frac{n-2b^{1/c}\sum_{i=1}^{n}\frac{1}{{\left(x_{i}-a\right)}^{1/c}+b^{1/c}}}{b\cdot c},\\ \frac{\partial LL(a,b,c)}{\partial c}= & \frac{-n\left(\ln b+c\right)+\sum_{i=1}^{n}\left[\ln\left(x_{i}-a\right)-2\cdot \frac{\ln\left(\frac{x_{i}-a}{b}\right)}{{\left(\frac{x_{i}-a}{b}\right)}^{1/c}+1}\right]}{c^{2}}. \end{array}$$

We employ the trust-region reflective optimization algorithm in both steps and provide the analytical Jacobian in the second one, except in a few cases where that Jacobian are initially ill-defined (this occurs when the variance is large). The tolerance for both searches is set to $10^{-9}$, both in the value of the function to be optimized and in the displacement to be performed on the search space at each optimization step.

## 5. Goodness-of-Fit Tests

In the literature, we can find a significant number of goodness-of-fit methods based on statistical hypothesis testing [35]. In general, these methods first calculate a given statistic from the sample at hand, based on the assumption that the sample comes from a certain theoretical distribution ($H_{0}$). Then they evaluate the probability that the statistic takes the calculated value or a larger one if the assumption is true—a right-tail test; if that probability falls below a given significance level α or, in other words, if the statistic value is equal or larger than a certain threshold $\tau_{\alpha }$, then the null hypothesis can be rejected.

Elsewhere we have shown the good performance of the Anderson–Darling statistic in the case of the *Log-logistic* distribution, with it being one of the best marginal models for round-trip times in networked robot settings [10]. However, we do not use it here due to the limitations discussed in the following section.

Recall that the *Exponential* distribution can produce values that fall exactly at the location with non-zero probability. Although the theoretical *Log-normal* and *Log-logistic* distributions cannot do that, it is possible in practice to obtain values exactly equal to the location of those distributions due to numerical errors and the discretization of round-trip times. As shown below, the statistic to be computed for the Anderson–Darling test cannot handle those cases well (singularities occur), and that produces failed tests. In this paper we use the Cramér-von Mises statistic; with only slightly less power, it deals naturally with that issue and thus improves the applicability of the test.

Both the Anderson–Darling and Cramér-von Mises tests belong to the class of the so-called EDF statistics, which are based on computing the experimental distribution function (EDF) of the sample and then testing the difference with a theoretical distribution. In particular, they follow the pattern for a general quadratic statistic defined as

(12) $$Q=n\int_{-\infty }^{\infty }{\left(F_{n}\left(x\right)-F\left(x\right)\right)}^{2}\psi \left(x\right)dF\left(x\right),$$

where $F_{n}\left(x\right)$ is the experimental distribution function, F(x) the theoretical one, and ψ(x) is a weighting function.

The Anderson–Darling and Cramér-von Mises tests instantiate the weighting function ψ(x) differently in order to obtain the so-called $A^{2}$ and $W^{2}$ statistics, respectively, where

(13) $$\begin{array}{cc} A^{2}\Rightarrow \psi \left(x\right) & =\frac{1}{F(x)\cdot (1-F(x))},\\ W^{2}\Rightarrow \psi \left(x\right) & =1. \end{array}$$

Note that the Cramér-von Mises test gives the same importance (weight) to all the parts of the support of the variable, while for the Anderson–Darling test, the tails, either where F(x)→0 or F(x)→1, are more important.

To derive the specific expressions for these statistics, we begin by applying the Probability Integral Transform; if F(x) is the true (continuous) distribution function of the data, then Z=F(X); i.e., the result of applying that function to the sample should follow a standard *uniform* distribution U(z;0,1). Furthermore, it turns out [35] that the differences F(x)−Z equal the ones required by Equation (12), $F_{n}\left(x\right)-F\left(x\right)$. Applying this result, if we arrange *Z* in increasing order, i.e., $Z=\langle z_{1},z_{2},\ldots ,z_{n}\rangle$, the following statistics are obtained:

(14) $$\begin{array}{cc} A^{2} & =-n-\frac{1}{n}\sum_{i=1}^{n}\left(2i-1\right)\left[ln\left(z_{i}\right)+ln\left(1-z_{n+1-i}\right)\right],\\ W^{2} & =\frac{1}{12n}+\sum_{i=1}^{n}{\left(z_{i}-\frac{2i-1}{2n}\right)}^{2}, \end{array}$$

where it can be observed that the suitability of is $W^{2}$ for cases where some $z_{i}$ is 0 or 1, unlike $A^{2}$.

If the sample actually comes from the proposed theoretical distribution (i.e., if $H_{0}$ holds) and that distribution is completely known in advance, that is, no parameter estimation is needed, then *Z* will certainly follow the cdf of U(z;0,1), a straight line with a slope of 1 and an intercept of 0; otherwise, when the parameters of the distribution are estimated from the very same sample, *Z* will no longer be *uniform*, and the distribution of the statistic ($W^{2}$) can have, in general, any arbitrarily complex shape; it will depend on the particular parameter estimation procedure, the sample size, and the theoretical distribution being tested.

There exist numerical tables for this kind of statistic, for which in some cases, Equation (14) has to be slightly modified, but since we use modified parameter estimation procedures in this work (Section 4), we need to provide new deductions of the thresholds for the tests. We do that through extensive Monte Carlo simulations starting with a minimum sample size of 20 up to a maximum size of 10,000.

The goodness-of-fit test for the *Exponential* distribution is the simplest one. We transform the sample *X* through the cdf of Equation (2) and then calculate $W^{2}$ from Equation (14). In [35] tables are provided for the thresholds to check. In Table 1 we collect the modifications of $W^{2}$ and the thresholds to use for the case of α=0.05, which we confirmed in our Monte Carlo experiments.

The tests for the *Log-normal* and *Log-logistic* distributions are the same as in [35] for the case of not estimating the parameters from the sample; we transform the data into another sample that is assumed to be drawn from U(z;0,1) by means of the Probability Integral Transform and then use the definition of the $W^{2}$ statistic in Equation (14) and the first row of Table 1 to complete the tests. The procedure is as follows:

- In the case of the *Log-normal* distribution, we transform the data into N(y;μ,σ) through $Y=\{ln\left(x_{i}-\gamma \right)\}$, then into a standard *normal* distribution through V=(Y−μ)/σ, and then apply the corresponding cdf, i.e., Z=Φ(V).
- In the case of the *Log-logistic* distribution, we first transform the sample into a non-located *Logistic* distribution with $Y=\{ln\left(x_{i}-a\right)\}$, then apply the cdf of the *Logistic* distribution, which is $F\left(y;b,c\right)=1/(1+exp\left(-\left(y_{i}-ln\left(b\right)\right)/c\right))$. Another common formulation for the *Logistic* distribution takes μ=ln(b) and σ=c as parameters.

However, since we proposed variations in the MLE procedures when the parameters are estimated from the sample in this paper (the semi-biased variation in the *Exponential* estimators, the extension of the Cohen’s method for the *Log-normal* distribution in Algorithm 1, and the two-stage non-linear optimization procedure with particular bounds on the allowed values of the parameters for the *Log-logistic* distribution), we need new mappings of the thresholds.

For that purpose, we processed a number of real experiments, which are publicly available at Zenodo [16], that contain sequences of round-trip times measured in a diversity of real environments where a remote computer requests sensory data from typical robotic sensors. This dataset contains sequences of RTTs, which are defined as in this paper, that involve communications between continents or local (same computer), transmissions of amounts of sensory data that are very diverse (from a few bytes to complete color camera snapshots), different operating systems and applications both in the client and server sides, and also widely different computer power (from simple embedded microcontrollers to high-end PCs); therefore we consider them to be quite representative of the diversity of situations that can be found in the context of networked robots.

We scanned all these time series by defining a window of a certain length *w* that moves along each round-trip-time sequence with increments of 10 at each step; for each window placement, we collect the corresponding round-trip-time sample and carry out parameter estimation on it for the three distributions defined in Section 3 using the procedures of Section 4.

Once all experiments were scanned with window sizes w∈[20,200], the estimated parameters for the considered distributions are collected, and the lower and upper bounds are determined for each of them. We consequently consider those bounds highly representative of very diverse situations of networked robots, and they are therefore suitable for carrying Monte Carlo simulations that draw values uniformly from the intervals they define. Table 2 lists these bounds. Actually, the distributions we build using those bounds cover much more cases than the ones likely producing the dataset, since we use all possible combinations of the parameters within the given ranges freely, without any restriction.

We estimate the thresholds $\tau_{\alpha }$ for the goodness-of-fit tests of the *Log-normal* and *Log-logistic* distributions by generating random distributions of those types using the mentioned bounds, drawing a number of samples of each given size from them, and then collecting the statistic $W^{2}$ that the tests would produce in that case. Note that we use the modifications suggested in [35] when all parameters are estimated from the sample: ${\bar{W}}^{2}=W^{2}\cdot \left(1+0.5/n\right)$ for the *Log-normal* distribution and ${\bar{W}}^{2}=\left(nW^{2}-0.08\right)/\left(n-1\right)$ for the *Log-logistic* distribution.

**Figure 2:**
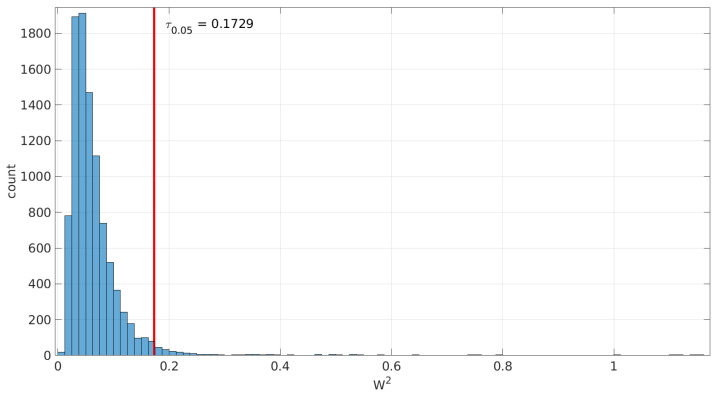
Monte Carlo simulation of 10,000 samples of size 200 drawn from random *Log-normal* distributions; the histogram is the distribution of the corresponding ${\bar{W}}^{2}$ statistic for the case of estimating all parameters of the distribution from the sample, while the red line marks the threshold $\tau_{0.05}$ that leaves 95% of the area to its left.

Since the samples are generated from the distribution to be tested, $H_{0}$ holds; therefore, we can use the histograms of the collected statistics ${\bar{W}}^{2}$ to deduce the threshold needed to decide a rejection under a given significance level (α) and sample size: that threshold has to leave to its right that area. For that purpose, the 1−α quantile of the collected statistic values is computed. Figure 2 illustrates an example of the obtained threshold for the goodness-of-fit test of the *Log-normal* distribution with a sample size of 200.

When this process is repeated for a number of sample sizes, we obtain a threshold ($\tau_{\alpha }$) for each one; plotting all of them together versus the sample size reveals the pattern to be used in the test.

**Figure 3:**
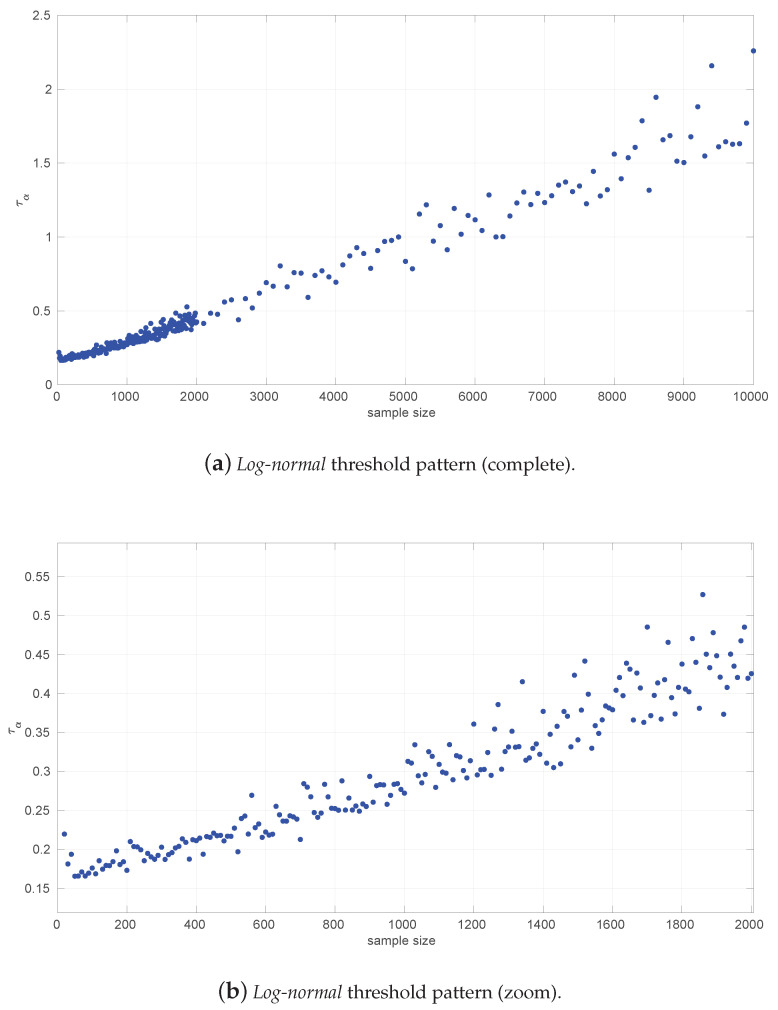
Threshold patterns to be used in the $W^{2}$ test for the *Log-normal* distribution. For each sample size (horizontal axis), the graph shows the estimated threshold to be used by the test in order to obtain a significance of 0.05, as explained in the main text.

**Figure 4:**
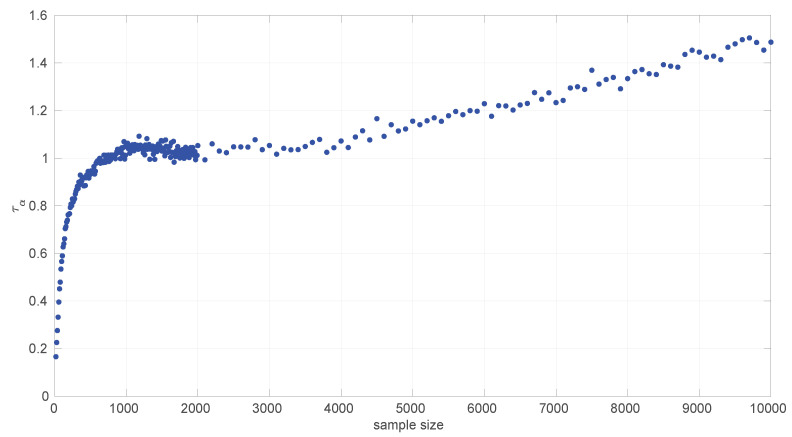
Threshold patterns for $\tau_{0.05}$ to be used in the goodness-of-fit $W^{2}$ test in the case of estimating the parameters of the *Log-logistic* distribution from the very same sample.

Figure 3 and Figure 4 show the resulting threshold patterns for both the *Log-normal* and *Log-logistic* distributions when considering α=0.05 and sample sizes in the interval [20, 10,000]. We carried out the Monte Carlo simulation for finer resolutions of sample sizes in the range [20,2000] because those sizes are expected to occur more frequently when modeling this kind of time series. Notice the higher variance in the thresholds for the *Log-normal* case, likely due to the higher variance in the estimation of parameters through the method of moments versus MLE.

The execution of these experiments is computationally intensive. To reduce the overall time employed in completing all the experiments, we distributed them among several machines and run them in parallel:

- *IMECH*: A high-performance workstation equipped with 4 NVIDIA RTX A6000 48 GB GPUs and an Intel Xeon Gold 5317 processor featuring 48 threads. It is provided by the Institute of Mechatronic Engineering & Cyber-Physical Systems [45] of the University of Málaga.
- *darkstar*: A high-performance workstation equipped with one NVIDIA RTX 5070 Ti 16 GB GPU and an AMD Ryzen 7 9800X3D processor featuring 16 threads.
- *garthim2*: A desktop computer equipped with an NVIDIA GeForce GT 710 GPU and an Intel(R) Core(TM) i9-10900KF CPU, offering 20 threads.

In order to use the patterns of Figure 3 and Figure 4 efficiently in practical implementations of goodness-of-fit tests, it is convenient to find analytical forms for them. Due to the different shapes that are shown in the figures for different parts of the sample size space and the need to provide a formula that minimizes the error with respect to the actual measured thresholds, we partitioned the data and fitted a different curve $f_{i}\left(s\right)$ to each part through a non-linear search of the minimum mean square error; then, adjacent parts of the curves were welded pairwise using sigmoid weights σ(s;k,T).

More concretely, if we have two consecutive parts, $f_{1}\left(s\right)$ and $f_{2}\left(s\right)$, defined, respectively, in the intervals $\left[s_{1},T\right]$ and $\left[T,s_{2}\right]$, with $s_{1}<T<s_{2}$, we can calculate their welding at points $s\in [\left(s_{1}+T\right)/2,\left(s_{2}+T\right)/2]$ as

(15) $$\begin{array}{c} f\left(s\right)=\left(1-\sigma (s;k,T)\right)\cdot f_{1}\left(s\right)+\sigma \left(s;k,T\right)\cdot f_{2}\left(s\right). \end{array}$$

In the case of an *s* value that lies in the first half of the first part or in the last half of the last part, no welding is performed and the corresponding part is directly evaluated.

The sigmoid weights are defined as follows:

(16) $$\sigma \left(x;k,T\right)=\frac{1}{1+e^{-k(x-T)}},$$

where the function yields weights in [0,1], *x* is the particular sample size, *k* the smoothing constant, and *T* is the joint point (a certain value in the sample size space). The sigmoid yields 0.5 at the joint point, thus giving the same weight to both adjacent parts at that place. The constant *k* is calculated in order to reach some weight $\bar{w}$ (e.g., 0.8) at a distance $\bar{l}$ from the joint point equal to 1/5 of the length of the shortest part, causing a fading effect of all welding relatively close to each joint point; for that condition to hold, $k>-ln\left(1/\bar{w}-1\right)/\bar{l}$, according to the definition in Equation (16).

The partition of each threshold pattern was performed visually by looking for the best segments that fit the polynomials, i.e., the familiar 1st-, 2nd-., or 3rd-order shapes. After deciding the points at which such shapes would change substantially, the search for the best polynomial for each segment was conducted through MSE fitting by attempting to solve the trade-off based on the following criteria:

- Small fitting residuals were usually improved by increasing the degree of the polynomial, but only to a point before producing overfitting.
- Fast calculations in their implementations were improved by decreasing the degree of the polynomial.
- The close placement of ending points results in the case where two polynomials have to be welded together.

On some occasions we repeated this search of a suitable trade-off after obtaining a complete welded solution because it was not completely satisfactory from a global perspective, i.e., considering all the segments at once.

**Figure 5:**
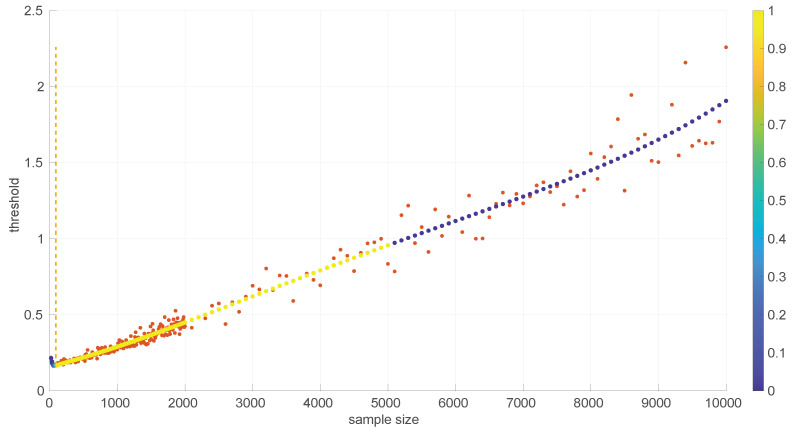
Analytical piecewise MSE fit of the *Log-normal* pattern of $\tau_{0.05}$ depicted in Figure 3. Two parts are modeled separately (marked with a vertical dashed line near the left) and welded smoothly at their joints. The color band on the right axis indicates the welding weight σ(s;k,T) used at each sample size; notice the abrupt changes in color at the middle of the second part, when the current pair of adjacent parts changes.

Figure 5 illustrates the resulting analytical form for the case of the *Log-normal* distribution. Two parts that can be fitted with the following polynomials and welded using $\bar{w}=0.8$ are distinguished:

(17) $$\begin{array}{cc} LN_{20:90}\left(s\right) & =-2.90535607380687\times 10^{-7}s^{3}+6.69820112318446e\times 10^{-5}s^{2}-\\ \; & 0.00505402498700543s+0.292511302536082,\\ LN_{90:10000}\left(s\right) & =2.38843364726652e\times 10^{-16}s^{4}-4.27021537891446e\times 10^{-12}s^{3}+\\ \; & 2.52914852901848e\times 10^{-8}s^{2}+0.000109960128411184s+0.159633019035599. \end{array}$$

Notice the very small values of some coefficients in some parts of Equation (17); we kept them because their presence improves the welding at the joint point, which is quite sensitive as discussed later on.

**Figure 6:**
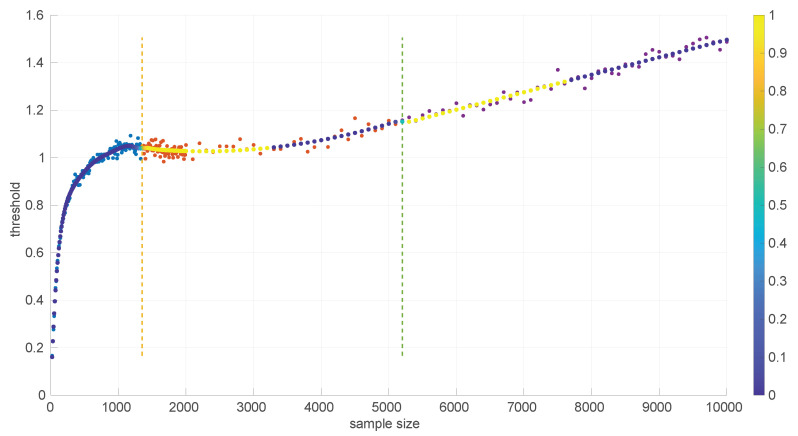
Analytical piecewise MSE fit of the *Log-logistic* pattern of $\tau_{0.05}$ where three parts were modeled separately (marked with vertical dashed lines) and welded smoothly at their joints.

A similar procedure was followed to provide an analytical form for the pattern of the threshold $\tau_{\alpha }$ in the case of the *Log-logistic* distribution. Figure 6 shows the resulting curve.

We distinguished three different parts here:

(18) $$\begin{array}{cc} LL_{20:1350}\left(s\right) & =8.47597347304412e\times 10^{-21}s^{7}-4.65037090157405e\times 10^{-17}s^{6}+\\ \; & 1.05107424918927e\times 10^{-13}s^{5}-1.26680627065562e\times 10^{-10}s^{4}+\\ \; & 8.80469214105822e\times 10^{-8}s^{3}-3.57199125291626e\times 10^{-5}s^{2}+\\ \; & 0.00830307365245381s+0.00800871978972110,\\ LL_{1350:5200}\left(s\right) & =1.54472346524334e\times 10^{-8}s^{2}-6.97828989559281e\times 10^{-5}s+1.10545573474545,\\ LL_{5200:10000} & =7.35465529012554e\times 10^{-5}s+0.760657621075085. \end{array}$$

The welding is conducted as in the *Log-normal* case but with $\bar{w}=0.999$.

**Figure 7:**
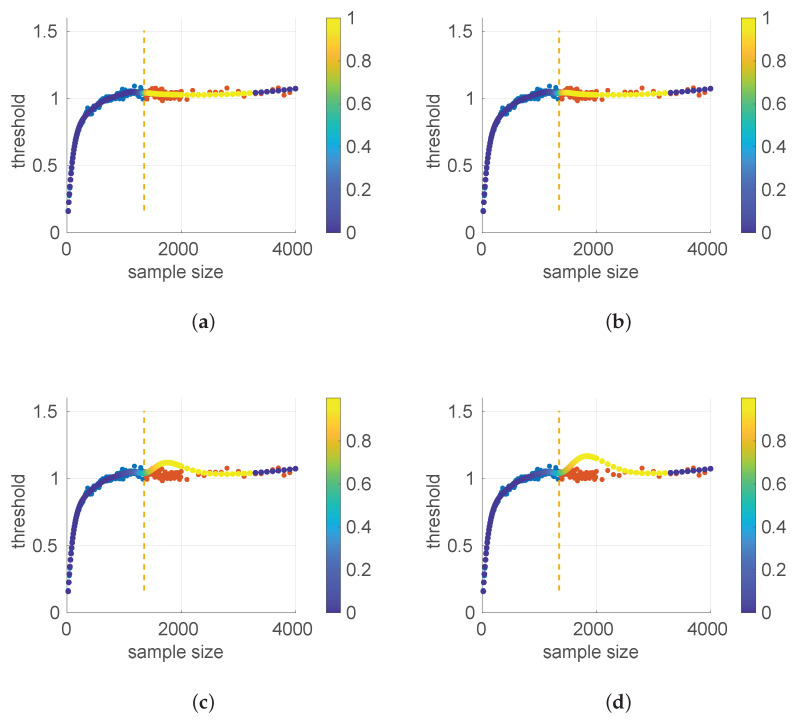
Sensitivity of the welding weight $\bar{w}$ for joining two of the polynomials of the approximation to the *Log-logistic* threshold pattern (see Figure 6). (**a**) $\bar{w}=0.999$, (**b**) $\bar{w}=0.99$, (**c**) $\bar{w}=0.9$, and (**d**) $\bar{w}=0.89$. The smaller its value, the more weight is assigned to the polynomial on the right as we go farther from the joint point.

We found that the sensitivity of the welding to the value of $\bar{w}$ is quite high; in Figure 7 we show the effect of reducing $\bar{w}$ in the first joint of the *Log-logistic* threshold approximation.

## 6. Evaluation

The first assessment of the proposed methods for modeling regimes in sequences of round-trip times is concerned with their power and significance. To test both, we conducted extensive Monte Carlo simulations of the distributions we defined in Section 3, taking their parameters uniformly from the intervals we identified in Table 2.

We also include in this section the study of the effects of the discretization of time measurements on the estimation procedures (Section 6.2).

Finally, we used the three models in all the scenarios of the previously mentioned dataset to find different regimes by gathering some measures that allow us to assess their utility and compare them.

### 6.1. Significance and Power

**Figure 8:**
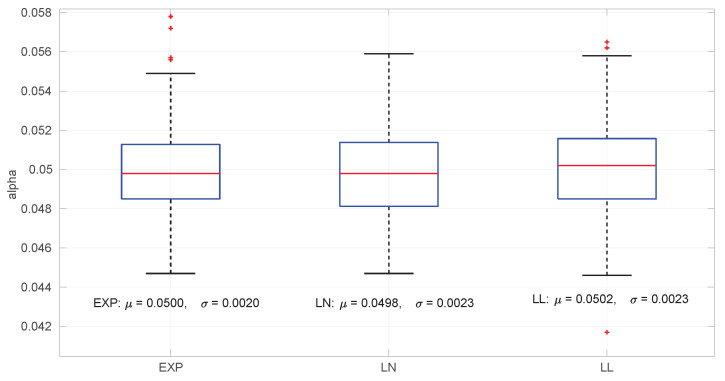
Monte Carlo estimation of the significance level for the goodness-of-fit tests of the probability distributions used in this paper. The expected significance level α is denoted as μ in the boxplots, and the error of its estimation (standard deviation) is denoted as σ.

For assessing the level of significance, we generated 10,000 random distributions of each type (*Exponential*, *Log-normal*, and *Log-logistic*) and drawn a sample from each one until a successful estimation of the distribution parameters is obtained for the sample; then we run the goodness-of-fit procedure described in Section 5 and counted how many times the test rejects the hypothesis $H_{0}$ (which we know is true), that is, the proportion of type I errors. The obtained expected α levels are very close to the designed 0.05, regardless of the sample size. Histograms of these α levels are shown in Figure 8.

On the other hand, the power of the tests is influenced by the sample sizes. We assessed this using several alternative hypotheses: *Exponential*, *Log-normal*, *Log-logistic*, *Trunc-normal*, and *uniform*. *Trunc-normal* has zero probability of producing values below certain locations (we cannot work with an infinite support in a networked robot) and has a normal shape beyond that point. For all of them we scanned sample sizes from 20 to 10,000 round-trip times and draw 10,000 samples randomly from the alternative distribution (randomly generated as well), ensuring that the parameters of the target distribution can be estimated, and then we counted the number of rejections of the null hypothesis.

**Figure 9:**
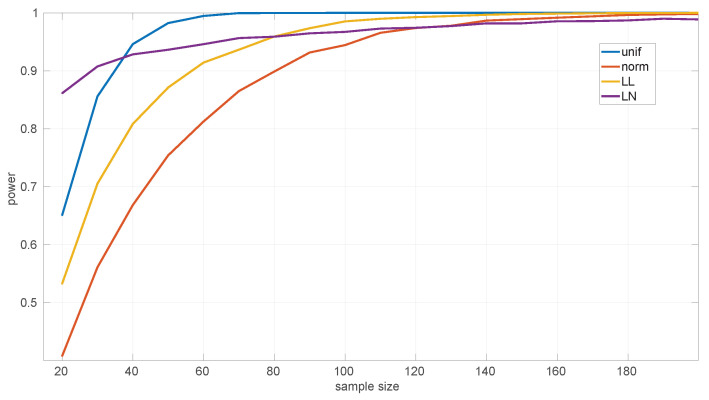
Monte Carlo estimation of the power of the *Exponential* goodness-of-fit test for different alternative hypotheses and diverse sample sizes. The missing portion until a sample size of 10,000 follows the same shape as in samples of size 200.

Figure 9 shows the results for the *Exponential* goodness-of-fit test. It can be seen that this test is more discriminative for *uniform* samples (a power of 0.9 from samples of size 35 onward) than for the *Log-logistic* (from 55 onward) and *Trunc-normal* (from 80 onward) samples; when the sample comes from a *Log-normal* distribution, the test shows a consistently high power, but not as high as for the other distributions in the case of large samples. The parameter estimation procedure for the *Exponential* distribution cannot fail.

**Figure 10:**
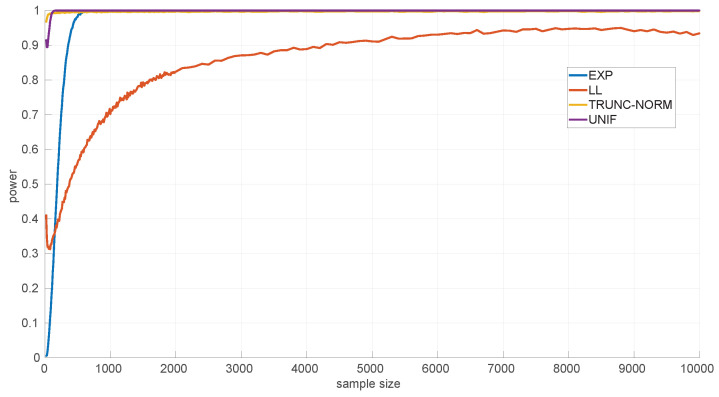
Monte Carlo estimation of the power of the *Log-normal* goodness-of-fit test for different alternative hypotheses and diverse sample sizes.

In Figure 10 we show the power for the *Log-normal* goodness-of-fit test. This test is highly discriminative for the *Trunc-normal* and *uniform* distribution, being >0.9 for all sample sizes, and less for the *Log-logistic* distribution, being >0.9 only from sample sizes of 4300 and up. This is mostly due to the natural closeness in shape of both distributions, but the non-negligible noise in the experimental thresholds of the *Log-normal* distribution can also have an effect. The practical consequence is that shorter *Log-logistic* samples will often be considered as *Log-normal* by this test. Designing a better procedure to estimate the thresholds (or to reduce the noise in their Monte Carlo calculation) is left as future work.

In our experiments, the procedure for estimating the parameters of the *Log-normal* distribution from the samples never failed to provide models to be assessed by the goodness-of-fit test.

**Figure 11:**
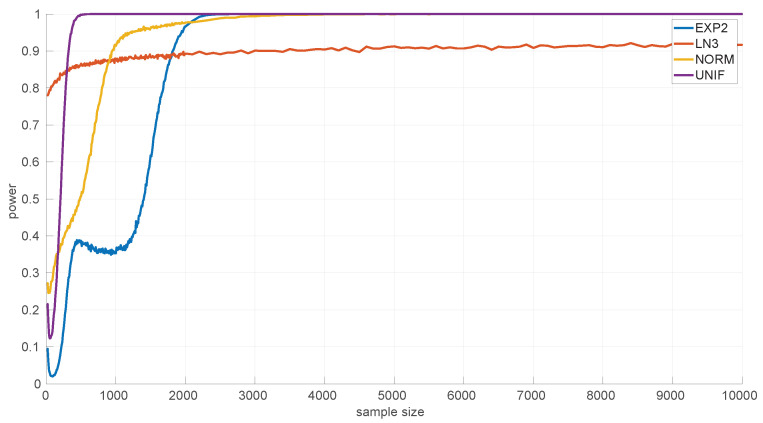
Monte Carlo estimation of the power of the *Log-logistic* goodness-of-fit test for different alternative hypotheses and diverse sample sizes.

Figure 11 shows the power curves for the *Log-logistic* distribution. The power of this test is similar to that of the *Log-normal* distribution but better with respect to that distribution (i.e., the *Log-logistic* distribution has more discriminative power for *Log-normal* samples than vice versa) and worse regarding the *Trunc-normal*, *uniform*, and *Exponential* distributions. The only abnormal issue is in this last case, where the power is kept low from sample sizes of [450,1200] approximately, which coincides with the first part of the threshold pattern curves in Figure 6. Therefore it is likely that the procedure to fit polynomials to those thresholds and/or the calculation of the thresholds itself through Monte Carlo could be improved. Nevertheless, the capabilities of the *Log-logistic* distribution for modeling and assessing regimes in sequences of RTTs is far superior to the ones of the other distributions, as we will see in Section 6.3.

As in the case of the *Log-normal* distribution, in our experiments, the procedure for estimating the parameters of the *Log-logistic* distribution from the samples never failed to provide models to be assessed by the goodness-of-fit test.

### 6.2. Effects of the Time Resolution on Parameter Estimation

As explained at the beginning of Section 4, round-trip times are measured as discrete values, while our models have continuous support. This may produce additional errors in our procedures, particularly during the estimation of parameters of the distribution corresponding to a given RTT sample. Note that in all the scenarios of the dataset used for our experiments, the time measurement resolution is of nanoseconds (a common clock resolution in modern CPUs), and when synthetic samples are drawn from distributions, the numeric resolution is set to ∼$10^{-16}$, i.e., up to 16 decimal digits; thus we avoid these errors in the experiments reported in other sections of the paper.

For a more in-depth analysis of the possible effects of having lower resolutions for time measurements, we generated 40 random samples from the three distributions described in Section 3 using parameters chosen also randomly from the ranges listed in Table 2; we then rounded the RTTs of each sample to a given resolution from a range covering from nanoseconds to milliseconds (which represents the vast majority of time-measuring systems that exist); finally we run the corresponding MLE procedure on the adjusted resolution sample. In this way, we can gather a statistical distribution of errors in the MLE-estimated parameters with respect to the true ones that produced the samples, as well as with respect to the ones that would be estimated with the maximum resolution (16 digits). We repeated the same process for each time resolution using sample sizes from 20 to 10,000 in steps of 100.

To maximize the generality of this analysis, the error metric should be relative to the magnitude of the true value of the parameter being estimated and also relative to the error obtained when using the maximum resolution. For that purpose, if $p_{i}$ is the true *i*-th parameter of the distribution that generated the sample, where ${\hat{p}}_{i}^{r}$ is its estimation with time resolution *r* and ${\hat{p}}_{i}^{max}$ is its estimation with the maximum time resolution, we calculate the normalized, relative error for that sample as follows:

(19) $$\begin{array}{cc} \delta^{max} & ={\hat{p}}_{i}^{max}-p_{i},\\ \delta^{r} & ={\hat{p}}_{i}^{r}-p_{i},\\ error & =\delta^{r}/\delta^{max}. \end{array}$$

According to this definition, a positive error indicates that the estimated parameter is larger than the true one, while a negative error indicates the opposite; the absolute magnitude of the error is the most important metric. Since we draw multiple samples, we obtain a statistical distribution for the error at the end.

The first conclusion of this study is that the sample size has no statistically significant influence on the error, in any of the models we analyze in this paper. This allows us to focus on the study of the effects of time resolution on a single sample size, which we have set to 100, and thus use a larger number of samples, namely 1000.

**Figure 12:**
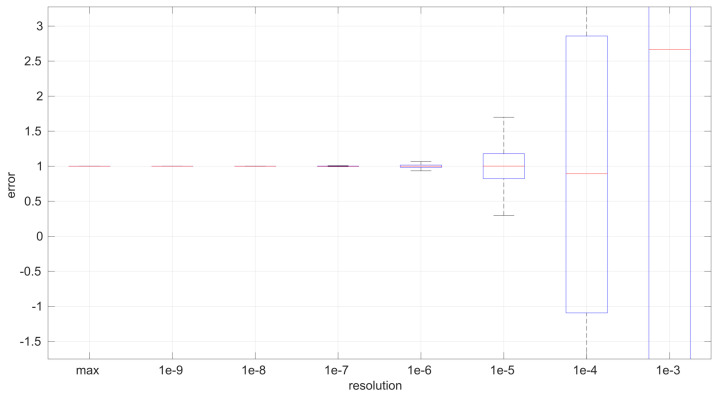
Effects of different time resolutions (horizontal axis, in seconds) on the error in estimating the α parameter of the *Exponential* distribution. We vertically zoomed out the graph, beyond the quartiles of the lowest resolutions (right-most boxes) in order to better appreciate the error size of the rest.

**Figure 13:**
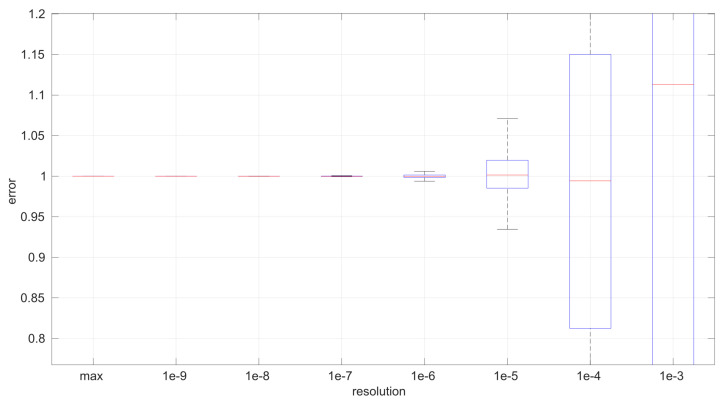
Effects of different time resolutions (horizontal axis, in seconds) on the error in estimating the β parameter of the *Exponential* distribution.

**Figure 14:**
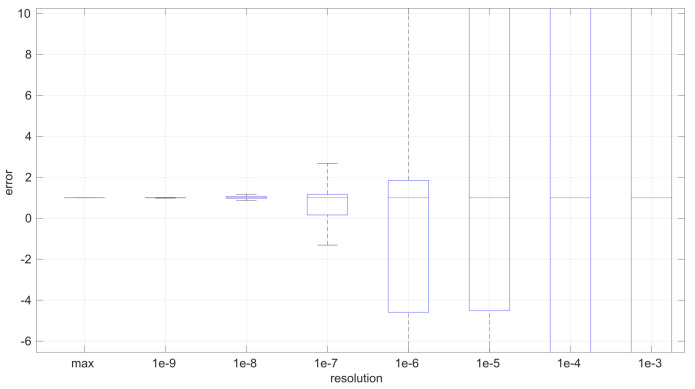
Effects of different time resolutions (horizontal axis, in seconds) on the error in estimating the γ parameter of the *Log-normal* distribution.

**Figure 15:**
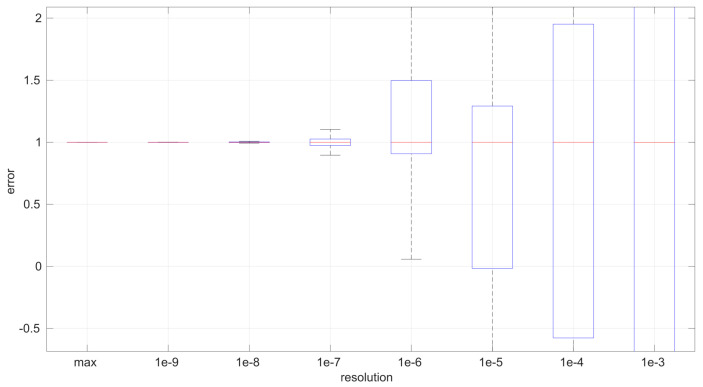
Effects of different time resolutions (horizontal axis, in seconds) on the error in estimating the μ parameter of the *Log-normal* distribution.

**Figure 16:**
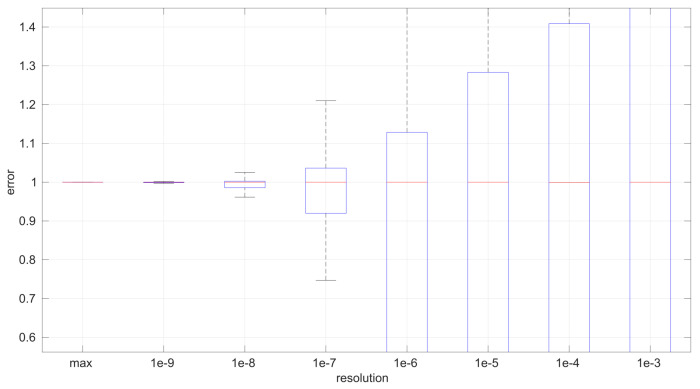
Effects of different time resolutions (horizontal axis, in seconds) on the error in estimating the σ parameter of the *Log-normal* distribution.

**Figure 17:**
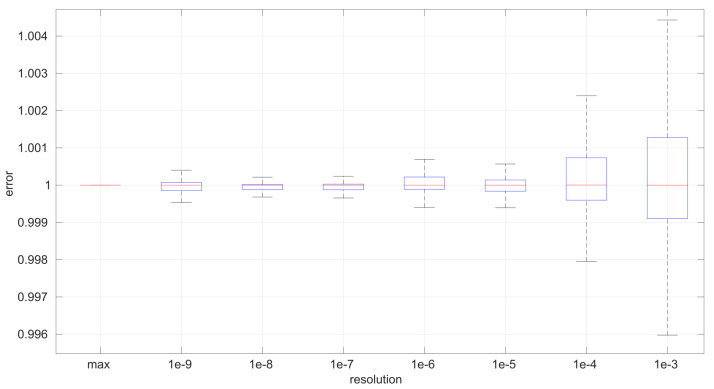
Effects of different time resolutions (horizontal axis, in seconds) on the error in estimating the *a* parameter of the *Log-logistic* distribution.

**Figure 18:**
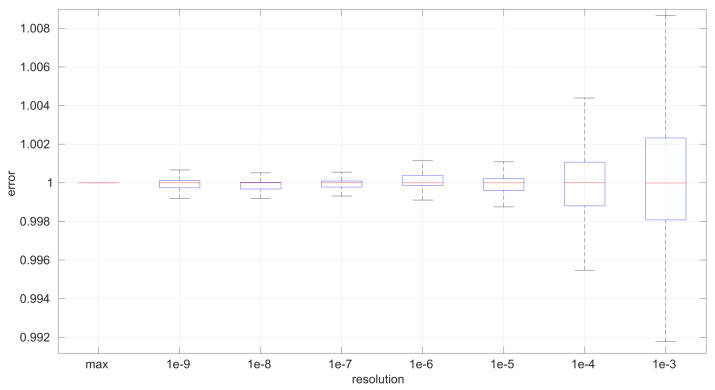
Effects of different time resolutions (horizontal axis, in seconds) on the error in estimating the *b* parameter of the *Log-logistic* distribution.

**Figure 19:**
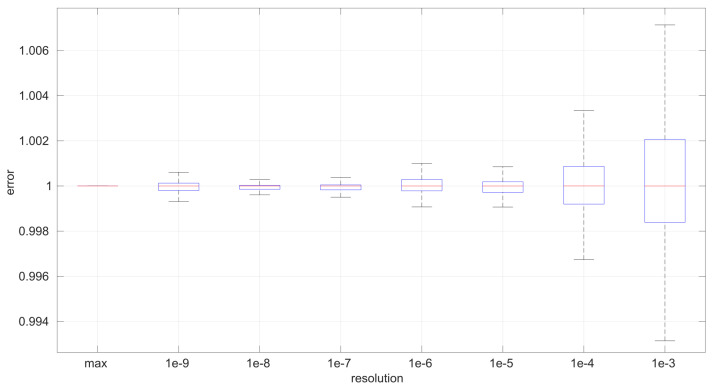
Effects of different time resolutions (horizontal axis, in seconds) on the error in estimating the *c* parameter of the *Log-logistic* distribution.

After calculating the normalized error per time resolution with a sample size of 100, it is clear that decreasing the time resolution produces more and more probability of having larger errors in the estimation, as shown in Figure 12, Figure 13, Figure 14, Figure 15, Figure 16, Figure 17, Figure 18 and Figure 19. The effect is particularly important in the *Exponential* and *Log-normal* distributions, with *Log-logistic* being much less sensitive to worse time resolutions. For a practical summary, the probability of having high errors becomes relevant when the resolutions become lower than the ones listed in Table 3. Notice that with a resolution of nanoseconds, which is common in modern OSes, there should be no special problems in those procedures.

### 6.3. Round-Trip Times Modeling in Networked Robot Scenarios

Besides assessing the significance and power of the three models and procedures proposed in this paper, we also evaluated their performance when modeling different regimes in the dataset of scenarios already introduced and described with more detail in [16].

**Figure 20:**
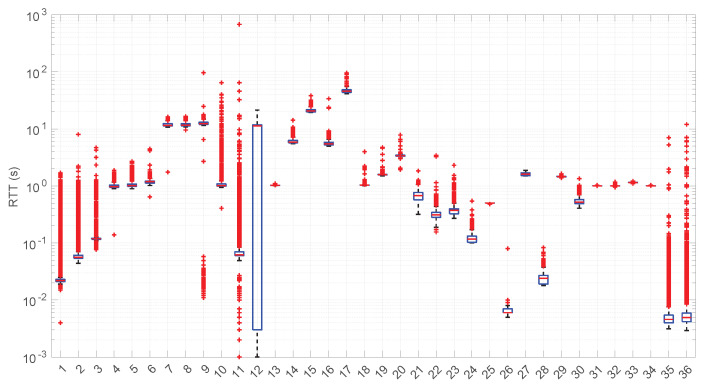
Summarized statistical description (medians, 1st and 3rd quartiles, and ±1.5 interquartiles and outliers) of the 36 scenarios of the dataset used in the paper. We used a logarithmic scale in the vertical axis. Notice the important right skewness of almost all scenarios, which calls for long- or heavy-tailed models.

In Figure 20, we summarize the statistical characteristics of the RTTs of all the scenarios of the dataset, where the skewness and long tails of their statistical distributions can be clearly appreciated, even with the logarithm scale used for a clearer visualization of the quartiles.

All the results that follow have been obtained based on all the scenarios of the dataset, but to explain some particularities of the methods, we have selected a few scenarios, as listed in Table 4. That table can serve as an index of the figures shown later on for those individual scenarios.

**Figure 21:**
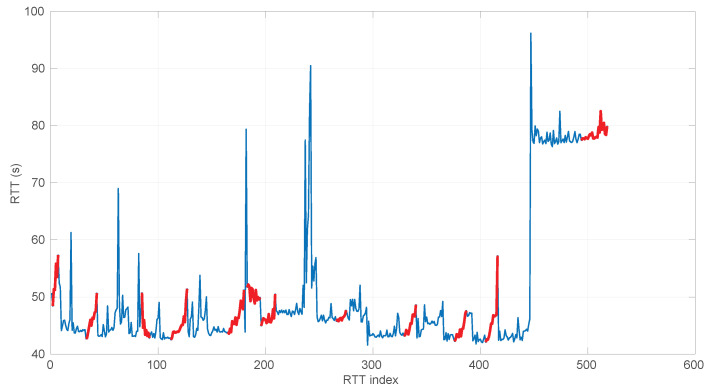
RTTs of scenario #17. It corresponds to a mobile robot sending a color camera picture through a network connection that includes a very slow RTC (phone) segment, which is in the same city as the client station; it runs Win32 OS in the robot and Linux in the client. We have highlighted in red some short trends (non-stationary segments) detected through visual inspection.

**Figure 22:**
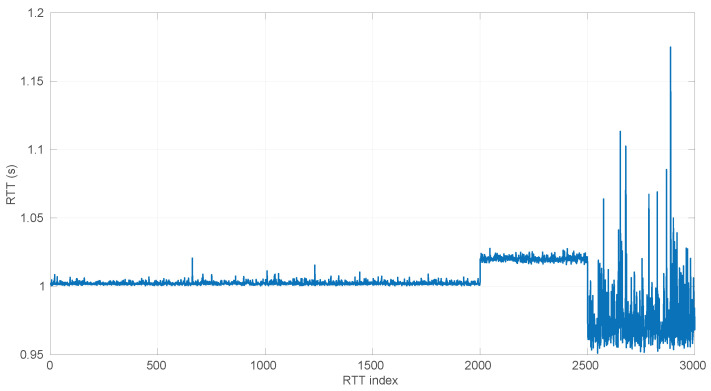
RTTs of scenario #32. This is a simulated scenario built by drawing samples from three different *Log-logistic* distributions with the parameters $a_{1}=1s$, $b_{1}=2$, $c_{1}=0.25$, $a_{2}=1.01s$, $b_{2}=10$, $c_{2}=0.1$, $a_{3}=0.95s$, $b_{3}=20$, and $c_{3}=0.4$. The 3 regimes are of different lengths and shapes.

**Figure 29:**
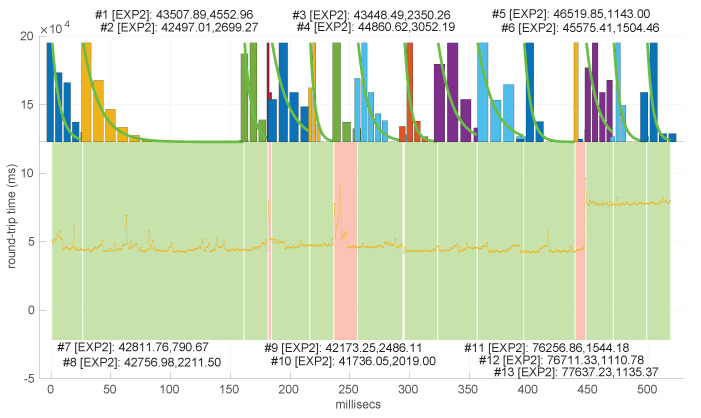
Regimes detected in scenario #17 with the *Exponential* procedures. Pink bands indicate unsuccessful modeling.

In Figure 21 and Figure 22, we show two scenarios in detail, where abrupt changes in regimes are visually very clear. In Figure 21, we also highlight some short trends appearing in the data of one of those scenarios. These effects are very rare—we have detected them only in three of our scenarios, and they are always very short. As it can be seen in the results of detecting regimes on this scenario with our three models (Figure 29, Figure 40 and Figure 47), in most of these situations, specifically when using the *Log-normal* and *Log-logistic* models, trends are absorbed in a given regime just like noise. The *Exponential* distribution separates the trend that can be observed at the end of the scenario from a previous regime; the *Log-normal* distribution breaks the current regime at approximately index #414 after the trend grows enough. In general, adapting the theoretical framework of marginal modeling to trended scenarios cannot be performed directly, since the marginal approach assumes *iid* RTTs or, in other words, entirely drops the dependencies produced by trends.

To apply our models to the scenarios in the dataset, we implemented a very simple procedure that just scans each scenario and looks for the longest regimes (sequences of consecutive RTTs sharing the same marginal model) that pass the goodness-of-fit tests. The pseudocode is in Algorithm 2. We have launched it for each distribution using a minimum regime length of 20 and limited the scenario lengths to a maximum of 10,000 round-trip times in order to finish the analysis in a reasonable amount of time. Our procedures are considered to be instantaneous from the point of view of the RTTs; i.e., their computation times do not alter those RTTs, though we have measured those computation times (which include estimating the parameters of the distribution—MLE fitting—plus running the goodness-of-fit test) to draw conclusions.

Recall that the characteristics of the CPU executing these experiments are listed in Section 5. However, now no parallelism is used—not even multi-threading—; thus the results are comparable to those that would be obtained in any common CPU. The procedures are written in sequential code in Matlab, except for the functions to be optimized in the *Log-logistic* MLE, which are written in C; the estimation of parameters of the distributions can actually benefit from parallelism, as we have reported elsewhere [46], but that is out of the scope of this paper.

In addition to measuring the computation time used by each distribution assessment, we also counted how many RTTs in a given sequence do our procedures need in order to finish their work, i.e., the batch size in an hypothetical batch (non-full) online modeling process. Recall that in this paper, we distinguish three cases concerning this as follows:

- *Offline estimation*, where the entire sequence of the RTTs must be gathered before providing a model for it.
- *Batch online estimation*, where the estimator can provide a useful model of a given segment of RTTs only after certain number of newer RTTs have been measured.
- *Full online estimation*, where the method can provide a model after each RTT is measured.

The number of RTTs that are gathered while we are still processing a previous one is a suitable metric for placing a method in some of these listed cases. Obviously, the ideal is the third one; i.e, the one that can provide a model after measuring a given RTT and before gathering the next one, but that is only (almost) completely achievable by the most simple model, the *Exponential* model, as we show later on.

From these regime modeling and change detection experiments, we can also gather other measures, namely those concerning the length of the detected regimes and the proportion of RTTs that are “explained” by the models, i.e., those that belong to some regimes that were successfully modeled and assessed.

In the next subsections, we discuss all the results.
**Algorithm 2** Detection of regimes in round-trip time sequences**function** DetectRegimes(*X*,δ,*s*)    $X=\langle x_{1},x_{2},\ldots ,x_{n}\rangle$ is the scenario, δ the distribution, s>0 the min. regime length    R←∅▹ list of regimes detected    l←∅▹ last model (regime) found    $f_{0}\leftarrow 1$▹ start index of the last model within *X*    **for** f∈〈1,n〉 **do**        **if** $f-f_{0}+1\geq s$ **then**           $m\leftarrow AssessModel(\langle x_{f_{0}},x_{f}\rangle ,\delta )$▹ MLE + GoF of segment $\left[f_{0},f\right]$ with distrib. δ           **if** m=∅ **then**▹ failed model with the new RTT $x_{f}$               **if** l≠∅ **then**▹ last model ended here                   $R\leftarrow R\cup (f_{0},f,l)$▹ new regime is built with last valid model                   l←∅▹ no last model                   $f_{0}\leftarrow f$▹$x_{f}$ has not been used for building last regime               **else**▹ no previous model; slide the window forward                   $f_{0}\leftarrow f_{0}+1$               **end if**           **else**▹ model assessed ok; go on extending it               l←m           **end if**        **end if**    **end for**    **if** l≠∅ **then**▹ trailing model        $R\leftarrow R\cup (f_{0},n,l)$    **end if**    return *R***end function**

### 6.4. Exponential Modeling

**Figure 23:**
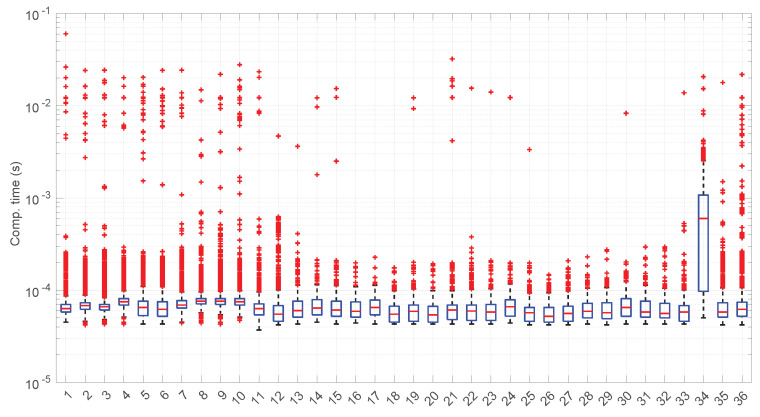
Computation time after reading each RTT value in the 36 scenarios of the dataset with the *Exponential* distribution. Note the logarithm scale in the ordinate axis. Most medians are around 60 ms.

Figure 23 shows the computation time taken by the parameter estimation and goodness-of-fit procedures at each step after measuring the current RTT when using the *Exponential* distribution. Marginal regime modeling and detection with this distribution allows for full online processing in the vast majority of scenarios (we omit the figure because its content can be very succinctly explained). However, there are a few exceptions: in scenarios #1, #12, #35, and #36 (among the “faster” ones), we found two steps where a maximum batch of four RTTs would be needed to finish modeling/assessments, three steps that need batches of three RTTs, and two steps that need batches of two RTTs. Since these situations are so rare in the dataset (we have processed a total of 38,958 RTTs from these four scenarios), we can consider the *Exponential* procedures to be suitable for full online processing, as they cause very infrequently short additional delays.

**Figure 24:**
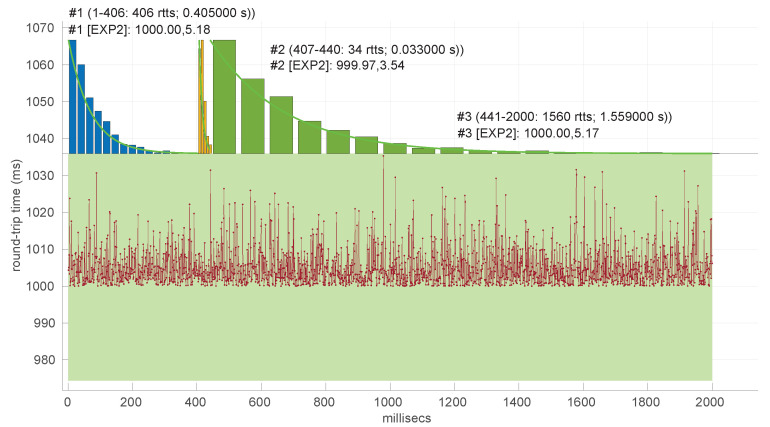
Regime detection with the *Exponential* procedures in scenario #34, a simulated one drawn from an *Exponential* distribution. Note that the third regime is virtually identical to the first one; thus the second can be considered a brief burst. In this and the rest of the figures showing regime detections, the bottom half is the sequence of round-trip times plotted at the moments they are gathered; the top half shows the numbered detected regimes (#1, #2, and #3) as histograms of those data, along with the mathematical forms (EXP2) and parameters of the models found for them (α and β) and the ranges of round-trip times (rtts) they model.

The most atypical situation for this distribution regarding its computational cost occurs in scenario #34 (Figure 24), where it takes around one order of magnitude more to compute than in the rest of the scenarios (interestingly enough, that scenario can be processed in a full online fashion). The reason the procedure takes longer is because it detects much longer regimes, and this is only natural, as shown in the figure—processing larger samples has a higher cost.

**Figure 25:**
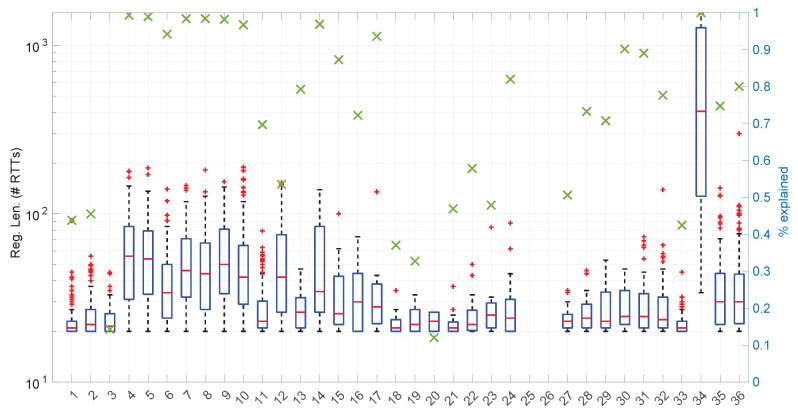
Regime lengths (boxplots, left axis) and percentage of explained RTTs (green crosses, right axis) after modeling and detecting changes in regimes in the 36 scenarios of the dataset with the *Exponential* distribution. Note the logarithm scale in the left axis.

Figure 25 shows the length of the regimes detected in each scenario with the *Exponential* distribution along with the percentage of RTTs explained by the *Exponential* model in each scenario (right axis). Both data indicate the affinity of the scenarios with the *Exponential* model. For instance, scenario #34 has very high affinity with the *Exponential* distribution, as commented before.

**Figure 26:**
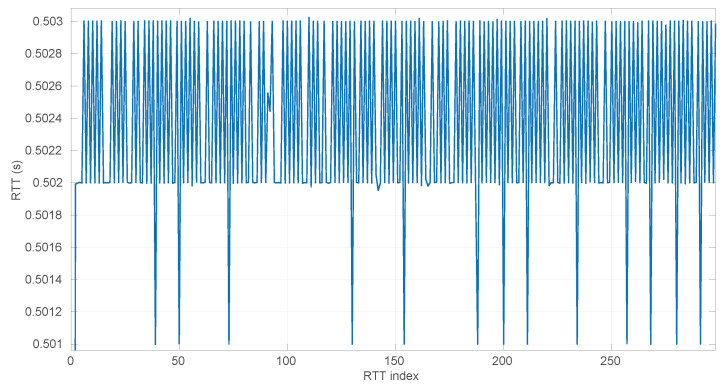
A portion at the beginning of scenario #25, where the *Exponential* procedures fail to assess any regime due to its high determinism.

**Figure 27:**
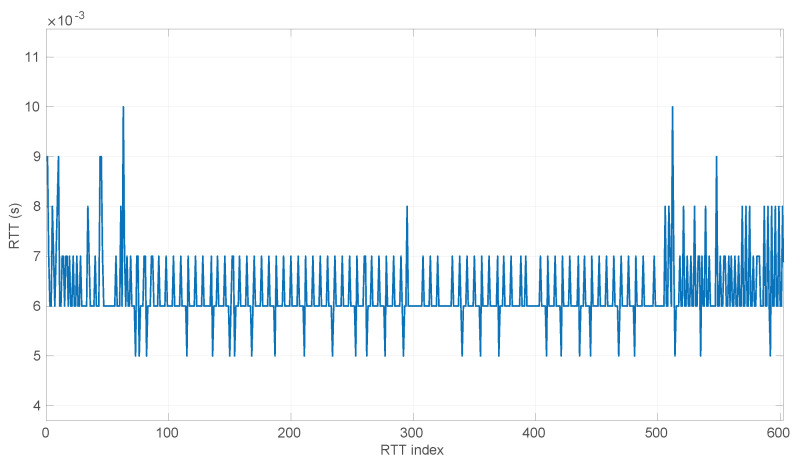
Part of scenario #26, highly deterministic like scenario #25 (Figure 26).

No regime is detected at all in scenarios #25 and #26 with the *Exponential* model. These two scenarios are detailed in Figure 26 and Figure 27. Their main particularity is their determinism, i.e., very low noise (recall Table 4).

**Figure 28:**
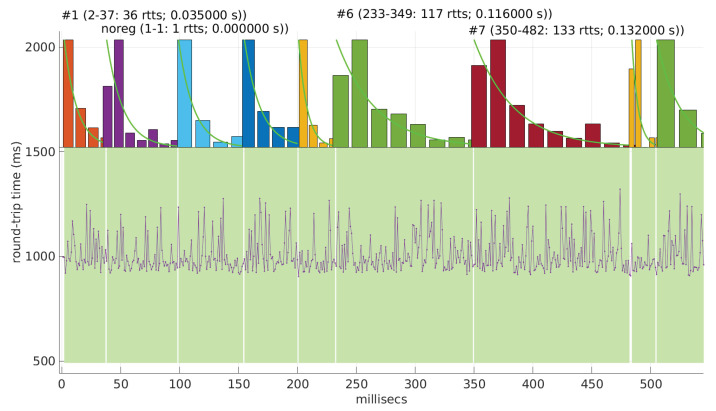
Some of the regimes detected at the beginning of scenario #4 with the *Exponential* procedures. The thin white separations in the bottom half of the figure are unexplained RTTs (labelled as “noreg”). The rest of the scenario has the same behavior as the one shown here—no visually detected changes in regimes or significant bursts or trends.

Finally, to obtain a practical grasp of the behaviour of this distribution when modeling regimes in real scenarios, Figure 28 shows its performance in the beginning of scenario #4, the one where it obtains longer regimes (leaving aside #34). Notice that in spite of detecting longer regimes and having a good coverage of the scenario (a high percentage of explained RTTs with the models, as shown in Figure 25), those regimes are very short and usually leave some RTTs unexplained in between.

**Figure 30:**
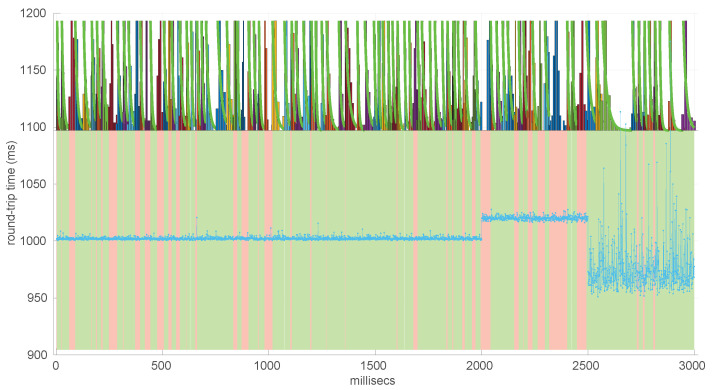
Regimes detected in scenario #32 with the *Exponential* procedures. There are so many (78) that we do not indicate their parameters for clarity.

Also, we have tested the behavior of this distribution against two of the scenarios that visually have clearer changes in regimes (#17 and #32), as commented before. Figure 29 and Figure 30 show the results. As it can be seen, the *Exponential* model is able to cover them, though not completely and with very short regimes. The detection of the abrupt change in regimes at time 447 ms of scenario #17 occurs right after reading that RTT (a previous unsuccessful—not passing the test—sequence of RTTs facilitates finding it so quickly). In the case of scenario #32, too many short regimes reflect the fact that the scenario does not come from an *Exponential* distribution; in that case, the first abrupt change, at time 2001 ms, is missed at 41 ms without successfully modeling the sequence, while the one at time 2501 ms is started before, at time 2493 ms, without detecting that the first RTTs belong to very different distributions.

### 6.5. Log-Normal Modeling

**Figure 31:**
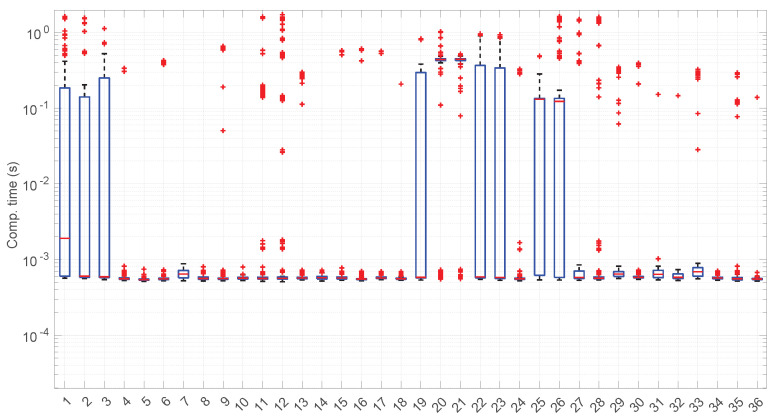
Computation time after reading each RTT value in the 36 scenarios of the dataset with the *Log-normal* distribution. Note the logarithm scale in the ordinate axis. Many medians are around ∼560 ms (zoomed in Figure 32).

**Figure 32:**
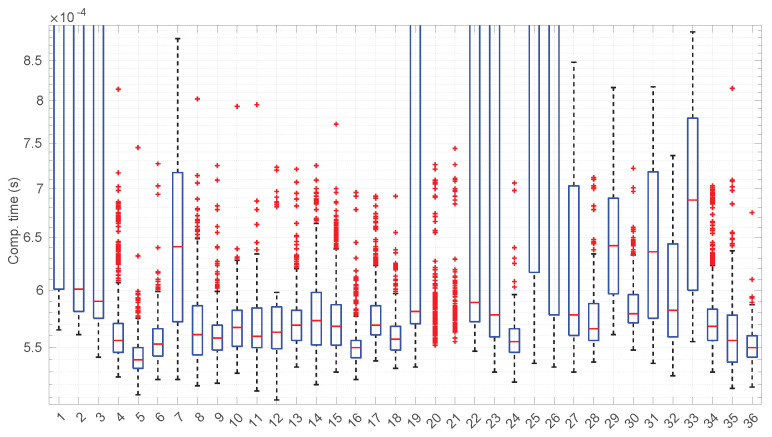
Zoom on the bottom of Figure 31 for a better appreciation of the scenarios where the computation is faster.

Figure 31 and Figure 32 show the computational time incurred by the *Log-normal* parameter estimation and goodness-of-fit procedures. These times are at least one order of magnitude longer than the ones of the *Exponential* model, reaching up to half a second in some cases. Using this distribution in a full online fashion has to be conducted with care.

**Figure 33:**
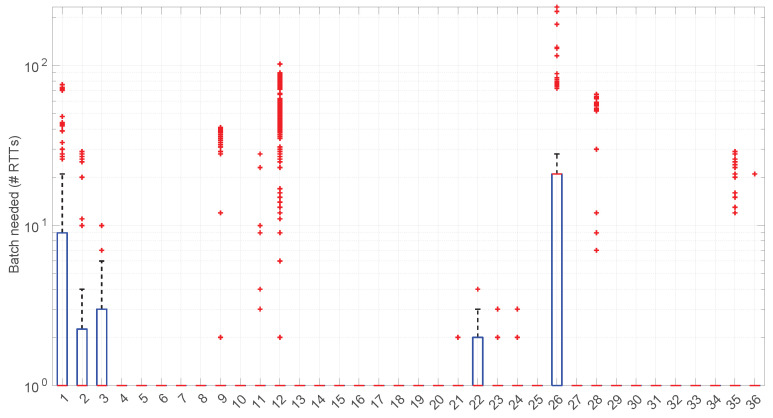
Number of additional RTTs passed before the computation of the *Log-normal* procedures finishes for the current RTT. Note the logarithm scale in the ordinate axis.

Figure 33 shows the batch size needed for the *Log-normal* procedures at each step of their work within each scenario. Many scenarios still allow for full online processing, but some of them need longer batches, up to a maximum of 20 (in median) or even ∼110 (maximum outlier, in scenario #26), in order to finish the processes.

Furthermore, a few scenarios with a median batch size of one, i.e., where non-batched, full online work could be possible, have outliers greater than one (they should use batches of ∼100 RTTs to not lose any round-trip measurement). All in all, 22 scenarios out of 36 admit full online processing.

**Figure 34:**
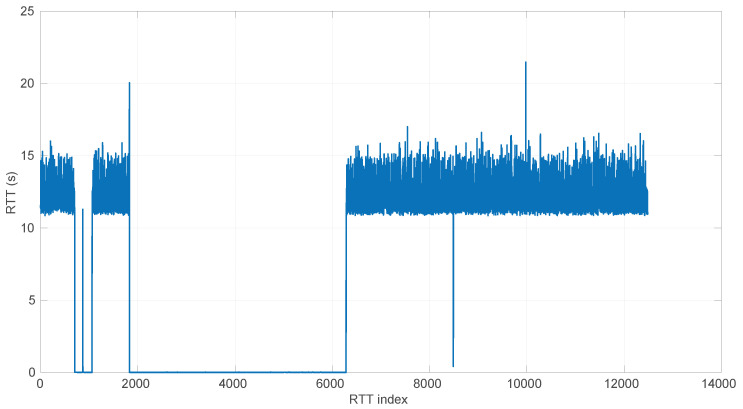
Scenario #12 causes problems for full online processing. In this scenario, a robot camera is sending 786,432 bytes of data from a linux server to a remote Windows laptop located in a different town of the same province through several optical fiber, twisted-pair, and wifi network segments. A zoom of the region approximately between indexes 1800 and 6500 is in Figure 35.

**Figure 35:**
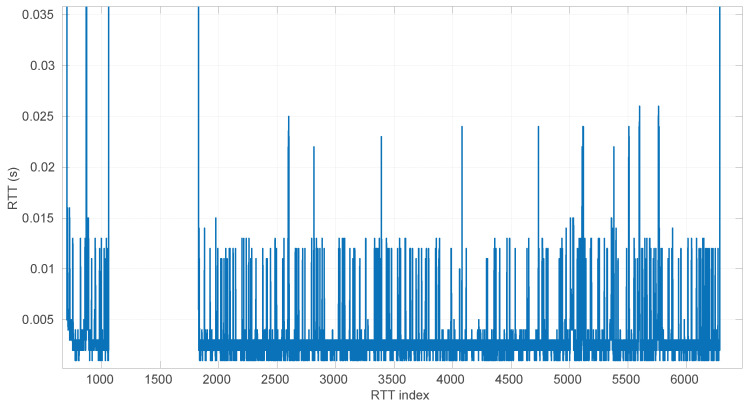
Zoom of the shortest RTT regimes in scenario #12. The abscissa axis shows the indexes of the RTTs instead of the time when they are gathered to better appreciate their number.

To illustrate this situation, scenario #12 is depicted in Figure 34. There it can be observed that the problem occurs in a number of RTTs that are very short with respect to the most frequent regimes (see the zoomed-in part of Figure 35). Scenario #26, which also present this need for long batches, has already been shown in Figure 27.

**Figure 36:**
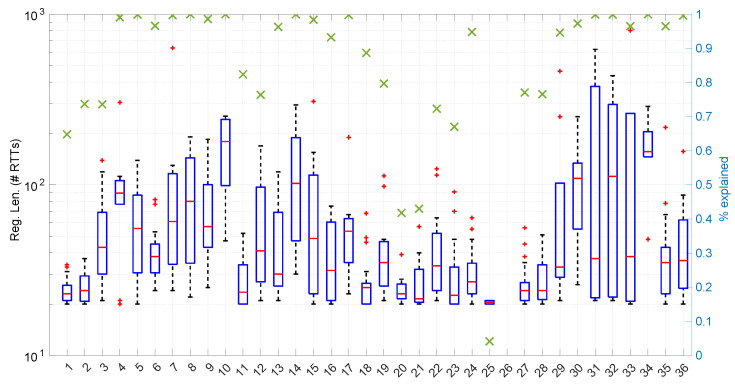
Regime lengths (boxplots, left axis) and percentage of explained RTTs (green crosses, right axis) after modeling and detecting changes in regimes in the 36 scenarios of the dataset with the *Log-normal* distribution. Note the logarithm scale in the left axis.

**Figure 37:**
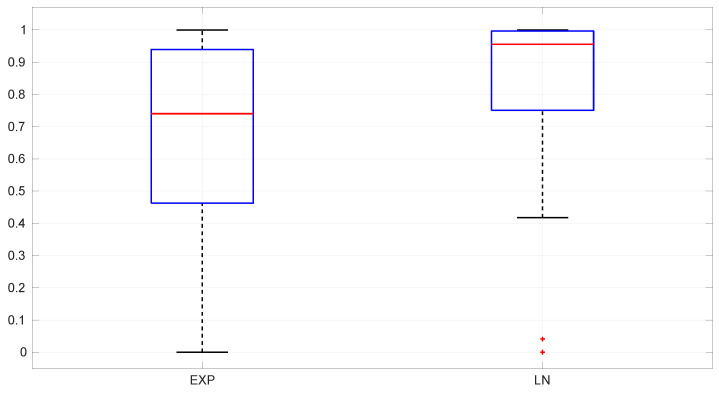
Statistical comparison of the percentage of explained RTTs of the *Exponential* and *Log-normal* distributions.

On the other hand, the *Log-normal* procedures provide a better explanation of the scenarios than the *Exponential* procedures, though with a similar regime length, as shown in Figure 36 and more clearly in Figure 37.

**Figure 38:**
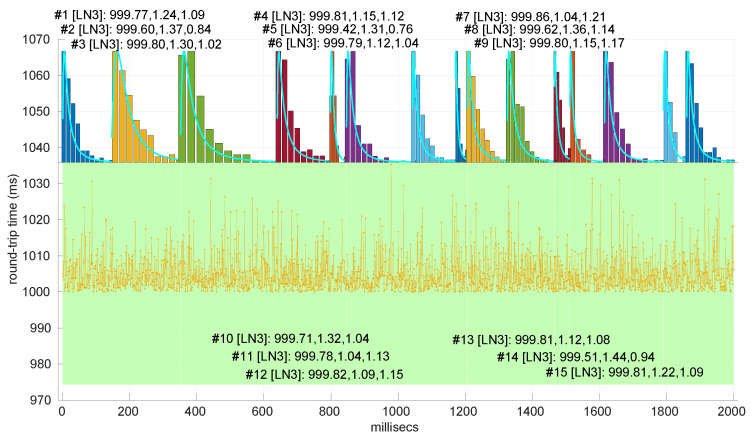
Regimes detected in scenario #34 with the *Log-normal* procedures compared with the result of applying the *Exponential* procedures to the same scenario (Figure 24).

**Figure 39:**
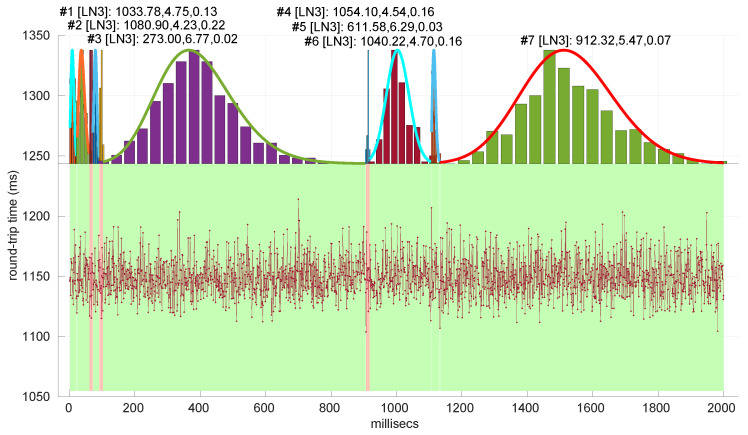
Regimes detected in scenario #33 with the *Log-normal* procedures. The pink bars in the bottom half are short sequences of RTTs that receive no assessed model.

Two examples of practical use of the *Log-normal* procedures are shown in Figure 38 (scenario #34, with the same shown for the *Exponential* procedures in Figure 24) and Figure 39 (scenario #33). In the former (recall that it is a simulated scenario drawn entirely from an *Exponential* distribution) the *Log-normal* model detects more regimes and is shorter than the *Exponential* model. The latter is also a simulated one and is generated from a *Log-normal* distribution that has a Gaussian-like shape. It, nevertheless, is recognized as several regimes because of some very short transient, non-modeled samples.

**Figure 40:**
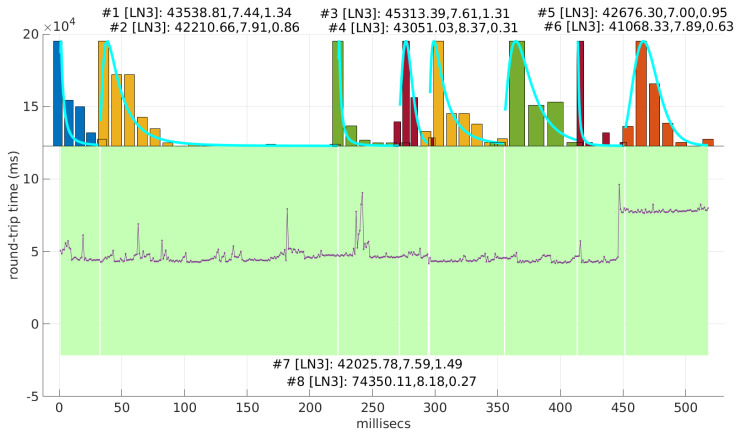
Regimes detected in scenario #17 with the *Log-normal* procedures.

**Figure 41:**
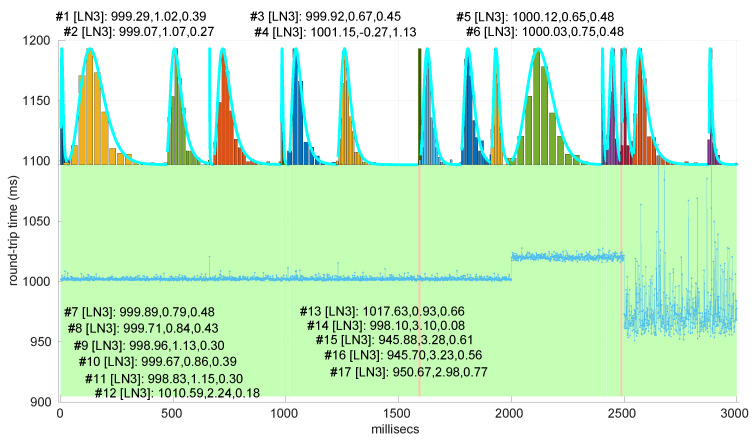
Regimes detected in scenario #32 with the *Log-normal* procedures.

We also tested the abrupt change in regime detection of this distribution against scenarios #17 and #32 (Figure 40 and Figure 41). The number of modeled regimes is less than that using the *Exponential* procedures, particularly in scenario #32, which is difficult to model, as explained before. The abrupt change in regimes at time 447 ms of scenario #17 is detected at time 452 ms, with just a short delay and without unsuccessful intermediate segments. In the case of scenario #32, the first abrupt change, at time 2001 ms, is detected immediately without delay (the previous regime does not support the inclusion of the first RTT that is different), while, as in the *Exponential* case, at time 2501 ms, the detected new regime starts before it should (at time 2493 ms).

### 6.6. Log-Logistic Modeling

**Figure 42:**
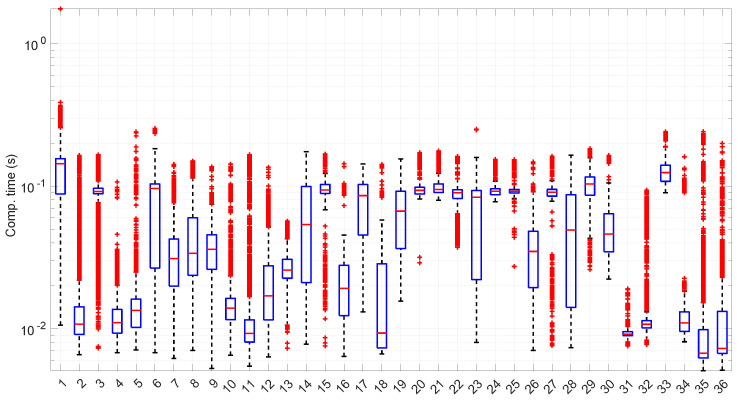
Computation time after reading each RTT value in the 36 scenarios of the dataset with the *Log-logistic* distribution. Note the logarithm scale in the ordinate axis. Here, the median computation times lie in the range [6, 200] ms.

The computation times per step of the *Log-logistic* procedures in all scenarios are collected in Figure 42. This distribution has a worse computational cost than the *Log-normal* distribution: around one order of magnitude slower in most scenarios and two if compared with the *Exponential* model. This is only natural due to the double non-linear optimization of the MLE parameter estimation procedure, but it has also a strong relation with the longer regimes assessed by this distribution.

**Figure 43:**
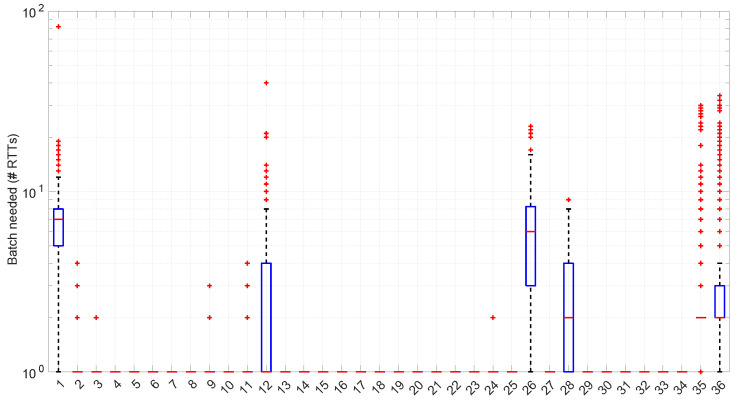
Number of additional RTTs passed before the computation of the *Log-logistic* procedures finishes for the current RTT. Note the logarithm scale in the ordinate axis.

The higher computational cost, however, does not worsen much the full online capabilities of the *Log-logistic* model with respect to the *Log-normal* model, as shown in Figure 43: now, 25 scenarios out of 36 can work with this distribution in a full online fashion, and the longer batch needed to assure no RTT is lost is ∼90, which is one order of magnitude smaller than the one for the *Log-normal* model.

**Figure 44:**
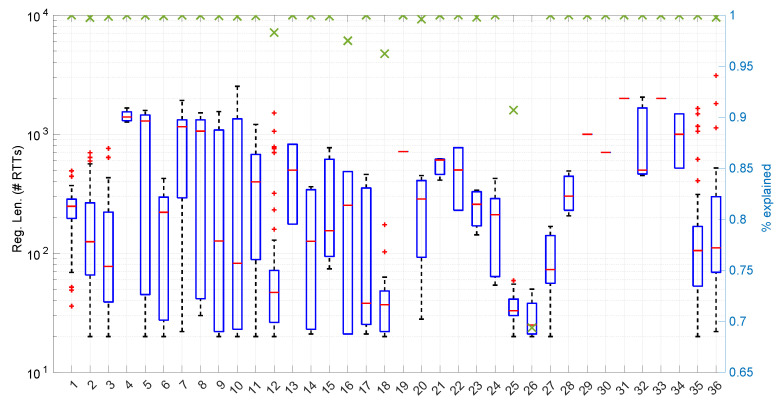
Regime lengths (boxplots, left axis) and percentage of explained RTTs (green crosses, right axis) after modeling and detecting changes in regimes in the 36 scenarios of the dataset with the *Log-logistic* distribution. Note that we have limited our experiments to scenarios of a maximum length of 10,000 RTTs.

**Figure 45:**
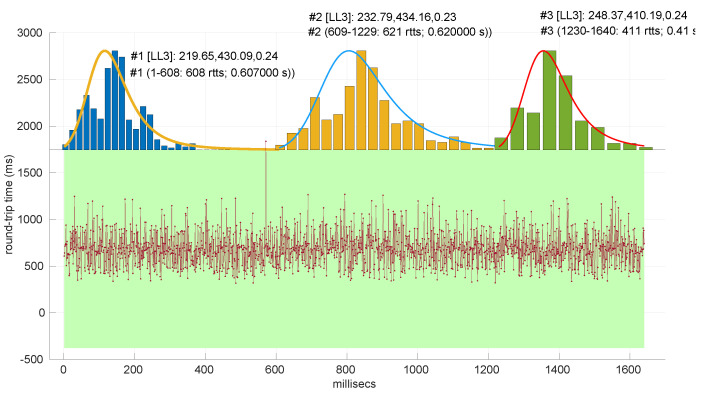
Regimes detected in scenario #21 with the *Log-logistic* procedures.

**Figure 46:**
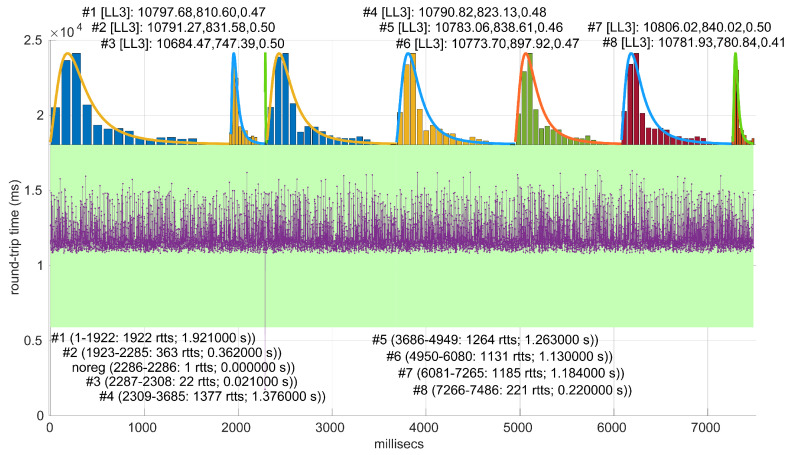
Regimes detected in scenario #7 with the *Log-logistic* procedures.

The *Log-logistic* model excels in how well it explain the scenarios, as shown in Figure 44: just one scenario has less than 0.7 coverage with this distribution, namely #26, which is one of the most deterministic ones (as shown in Figure 27); the reasons are the same as in the case of the *Exponential* and *Log-normal* distributions: having such a low level of noise prevents good models. The other three scenarios are covered in more than 0.9 with the *Log-logistic* model, and the vast majority obtain a complete coverage. Also, the regimes are quite long in many scenarios, as it has been shown in Figure 44. Examples of this are Figure 45 and Figure 46, which show the regimes detected in scenarios #21 and #7.

**Figure 47:**
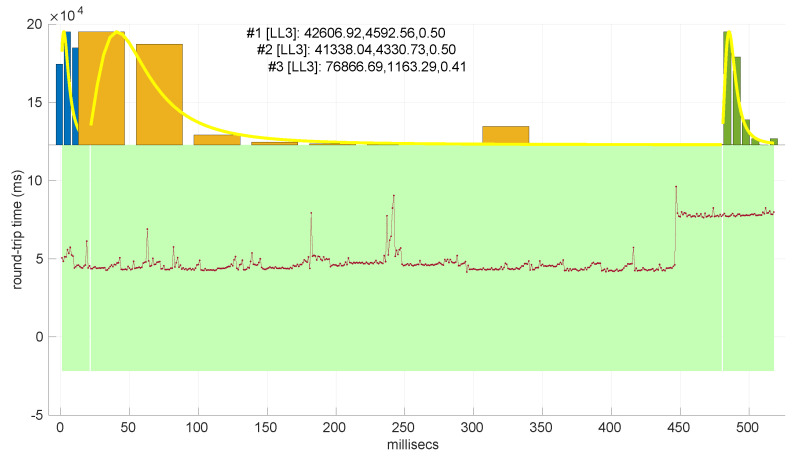
Regimes detected in scenario #17 with the *Log-logistic* procedures.

**Figure 48:**
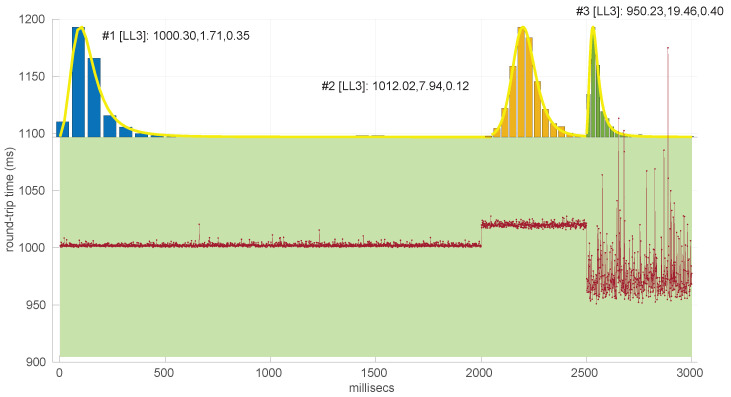
Regimes detected in scenario #32 with the *Log-logistic* procedures, showing an almost perfect match with the true distributions that generated the different regimes of the scenario.

We finally tested the abrupt change in regime detection of this distribution against scenarios #17 and #32 (Figure 47 and Figure 48). The number of modeled regimes is the smallest of the three distributions, and the lengths of those regimes are the largest. The abrupt change in regimes at time 447 ms of #17 is detected at time 481 ms, which is the longest delay in detection with respect to the other distributions; this is due to the flexibility of this one to keep a valid model with the first RTTs of the new regime. In the case of #32, the first abrupt change, at time 2001 ms, is detected at 2049 ms due to the same effect; however, the third regime is so different in shape to the second one that it is detected immediately (recall that this scenario is a synthetic one produced by *Log-logistic* distributions, whose true parameters are estimated very accurately). The greater flexibility of the *Log-logistic* mdoel should therefore be taken into account when considering it to detect regime changes quickly.

## 7. Conclusions and Future Work

In this paper we have studied in depth the use of marginal probabilistic distributions for modeling RTTs in the context of networked robot sensory transmissions, an approach that, in spite of dropping dependencies existing between consecutive RTTs, aims to reduce the computational cost with respect to other methods and, at the same time, provides as much useful information about the RTTs as possible, including not only the one needed to predict the next expected RTT but also any other deductions that can be drawn from a probability distribution, e.g., how likely it is that the next RTT lies in a given interval of values.

In that context of detecting abrupt changes in regimes, the usual gathered sequences of RTTs have no relevant trends—they include stationary noise, short bursts, and abrupt changes. Marginal modeling copes naturally with the noise, but it also easily leads to a statistically rigorous method for detecting abrupt changes: hypothesis testing, in particular, goodness-of-fit tests.

In this paper, we have focused on providing a thorough analysis of these tests for three particular distributions: *Exponential*, *Log-normal* and *Log-logistic*. All of them have been widely used before in the network communication community except the latter, which has only be applied, to the knowledge of the authors, in the particular context of networked robots.

Though the probabilistic models of those distributions are well known, even in the location forms we need, we add adjustments in the estimation of their parameters from samples of RTTs in order to improve their unbiasness and closeness to the desirable maximum likelihood estimation while taking into account possible numerical issues and computational costs. These adjustments require new tabulations of the thresholds used in the goodness-of-fit tests, which we have calculated through extensive Monte Carlo experiments. The result are novel piecewise polynomical curves that provide a convenient way of knowing the threshold for a given sample size.

We also assess the significance and power of the three procedures and conclude that each distribution has its own cons and pros regarding two important aspects: computational cost (the *Exponential* procedure is faster) and modeling capabilities of the scenarios (the *Log-logistic* procedure excels in that). In addition, we confirm whether these marginal models can be used in a full online fashion, i.e., whether the measuring of each RTT can be finished before the next RTT, or need a batch online strategy, i.e., work while several RTTs are collected to provide a result only after them (therefore injecting some delay in the modeling process). Both the *Log-normal* and *Log-logistic* models require a batch online strategy in a given proportion of scenarios. Another conclusion is that the greater flexibility of the *Log-logistic* distribution to model this kind of data has a disadvantage: it detects abrupt changes in regimes later because the new RTTs can still be explained by the last model for a while. Finally, we have explained that a certain amount of noise is necessary for these methods to work: if the RTT sequences are too deterministic, they fail to provide models.

All our experiments have been run on real (and a few simulated) scenarios gathered throughout the years and previously reported both in other papers and in their own Zenodo public repository, involving communications between continents or local (same computer), amounts of sensory data that are very diverse (from a few bytes to complete color camera snapshots), different operating systems and applications, and computer power (from simple embedded microcontrollers to high-end PCs); therefore, they are quite representative of the diversity of situations that can be found in the context of networked robots.

Our results confirm that marginal modeling is a suitable and robust approach not only to provide complete statistical information needed for the successful operation of networked robots (or other applications similarly distributed with non-deterministic round-trip times) but also to detect changes in the parameters of the RTTs quickly and rigorously.

In the future we plan to improve this study in several aspects: improving parameter estimation procedures (better adjusted to the actual data and therefore with more power), optimizing algebraic modeling of the thresholds needed for the tests (the current heuristic process could be investigated further in order to propose a more optimal and automated algorithm), focusing on sophisticated implementations of the methods in order to minimize their computational cost (e.g., through parallelization or by implementing incremental MLEs based on some kind of filtering, such as NLMs, which have had practical applications in the networking community [47]), designing procedures to successfully cache the assessed model for future regimes that could re-used them, and identifying their application to modeling and predicting RTTs in real robotic tasks, particularly those that require closed loops with certain timing requirements.

In addition, this research sets the basis for integrating in a rigorous mathematical model additional sources of knowledge and information—beyond the gathered RTTs and the chosen distribution—such as the network counters provided by Internet interfaces [48]; designing those integration procedures and evaluating the improvement in quality of the obtained models have been left as future work as well.

## Figures and Tables

**Table 1:** *Exponential* goodness-of-fit: ${\bar{W}}^{2}\geq \tau_{0.05}$ rejects $H_{0}$; i.e., the sample is unlikely to have been drawn from that *Exponential*.

Parameters	Statistic Modification	Threshold
Not estimated	${\bar{W}}^{2}=\left(W^{2}-0.4/n+0.6/n^{2}\right)\cdot \left(1+1/n\right)$	$\tau_{0.05}=0.461$
Estimated	${\bar{W}}^{2}=W^{2}\cdot \left(1+2.8/n-3/n^{2}\right)$	$\tau_{0.05}=0.222$

**Table 2:** Lower and upper bounds for the parameters of the different distributions when scanning a variety of real experiments. Values are rounded to cover wider intervals. Locations are in milliseconds.

Distribution	Lower Bounds	Upper Bounds
*Exponential*	$\alpha_{L}=10^{-4}$, $\beta_{L}=10^{-5}$	$\alpha_{U}=26\times 10^{3}$, $\beta_{U}=5$
*Log-normal*	$\gamma_{L}=10^{-4}$, $\mu_{L}=10^{-10}$, $\sigma_{L}=10^{-3}$	$\gamma_{U}=76\times 10^{3}$, $\mu_{U}=10$, $\sigma_{U}=8$
*Log-logistic*	$a_{L}=10^{-4}$, $b_{L}=10^{-4}$, $c_{L}=0.05$	$a_{U}=76\times 10^{3}$, $b_{U}=32\times 10^{3}$, $c_{U}=0.45$

**Table 3:** Time resolutions below which the proposed parameter estimation procedures suffer from significant errors.

Distribution	Parameters	Least Suitable Resolution
*Exponential*	α and β	tenths of microseconds
*Log-normal*	γ and σ	hundredths of microseconds
	μ	tenths of microseconds
*Log-logistic*	*a*, *b* and *c*	milliseconds

**Table 4:** Scenarios of the dataset on which we illustrate some particularities of the methods.

Scenario	Definition	Particularities
#4—Figure 28	On board the robot; serving 65,536 bytes of data; Linux PC; direct software socket connection.	A real scenario where the *Exponential* distribution detects longer regimes.
#7—Figure 46	On board the robot; serving 786,432 camera data; Linux PC; direct software socket connection.	Used to illustrate the good coverage of the data by the *Log-logistic* distribution.
#12—Figure 34 and Figure 35	Robot serving 786,432 bytes of camera data to a remote station in a different town; Linux client and Windows server; twisted pair + WiFi + fiber cable network segments.	Due to a regime with very short communication times, the fastest method (*Exponential*) cannot achieve full online processing.
#17—Figure 21, Figure 29, Figure 40 and Figure 47	Robot serving 921,600 bytes of camera data to a remote station in the same town; Linux client and Windows server; twisted pair + WiFi + RTC56Kbps old phone connection.	Exhibits a very clear abrupt change in regimes plus bursts and a number of much smaller changes.
#21—Figure 45	Small surveyor robot sending 230,400 bytes of its camera sensor data to a smartphone in the same building; Android OS in both devices; WiFi ad hoc network connection.	Same as for scenario #7.
#25—Figure 26	Arduino board sending 27 bytes of data from an analog sensor to a PC; Linux on the client and a baremetal C program in the server; USB connection.	Highly deterministic scenario.
#26—Figure 27	On board the robot, sending 4 bytes of data to a PC in the same building; Linux in the client and Windows in the server; twisted pair connection.	Same as scenario #25.
#32—Figure 22, Figure 30, Figure 41 and Figure 48	Synthetic scenario built by concatenating three *Log-logistic* regimes.	Contains pure abrupt changes in regimes; no bursts and no trends.
#33—Figure 39	Synthetic scenario generated by a single *Log-normal* distribution with the parameters γ=1s, μ=5, and σ=0.1.	No regime changes, bursts, or trends; a single, well-defined source of noise.
#34—Figure 24 and Figure 38	Synthetic scenario generated by a single *Exponential* distribution with the parameters α=1s and β=5.	No regime changes, bursts, or trends; a single, well-defined source of noise.

## Data Availability

The dataset on networked robot RTTs is publicly available at Zenodo [16]. The Matlab code we used to perform the analyses and obtain the results of this paper is also publicly available at Github.
